# Supplementary material for: Spatial patterns of microbial communities across surface waters of the Great Barrier Reef
Source: Commun Biol. 2020 Aug 14;3:442. doi: 10.1038/s42003-020-01166-y (PMC7428009; doi:10.1038/s42003-020-01166-y)
Supplement: Supplementary file 1 — Supplementary Information [file 42003_2020_1166_MOESM1_ESM.pdf]

Supplementary material

"Spatial patterns of microbial communities across surface waters of the Great Barrier Reef"

Frade et al.

Supplementary Figures

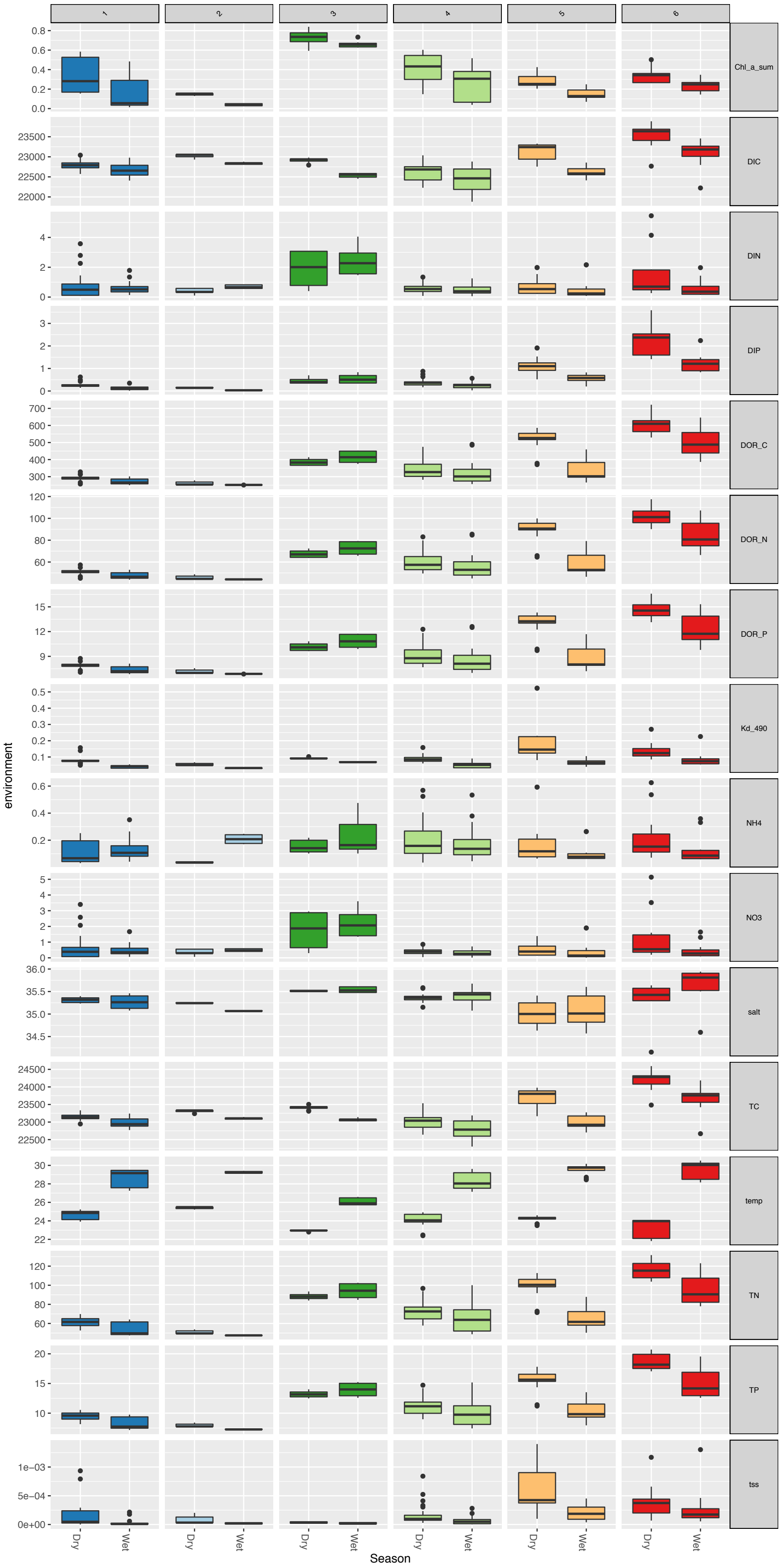

Supplementary Fig. 1A

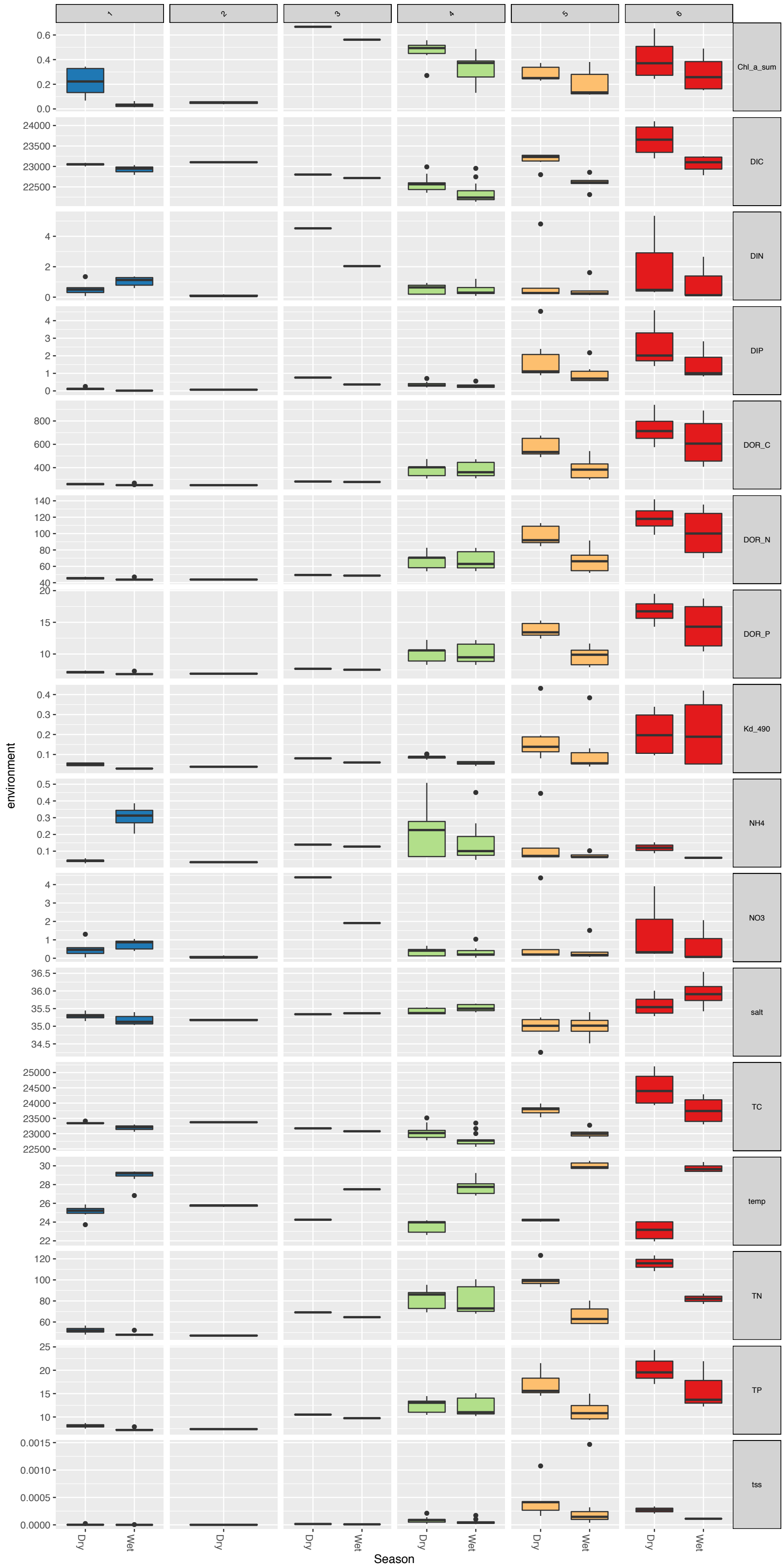

Supplementary Fig. 1B.

Supplementary Fig. 1. Cross-shelf and inter-seasonal environmental variation across the GBR for parameters retrieved from the eReefs platform for a) LTMP sites and b) microbial sites. Chl_a_sum: total chlorophyll *a* (mg m^{-3}), DIC: dissolved inorganic carbon (mg m^{-3}), DIN: dissolved inorganic nitrogen (mg m^{-3}), DIP: dissolved inorganic phosphorus (mg m^{-3}), DOR_C: dissolved organic carbon (mg m^{-3}), DOR_N: dissolved organic nitrogen (mg m^{-3}), DOR_P: dissolved organic phosphorus (mg m^{-3}), KD_490: vertical attenuation coefficient of light at 490nm (m^{-1}), NH4: ammonium (mg m^{-3}), NO3: nitrate (mg m^{-3}), salt: salinity (psu), TC: total carbon (mg m^{-3}), temp: temperature (°C), TN: total nitrogen (mg m^{-3}), TP: total phosphorus (mg m^{-3}), and tss (g m^{-3}): total suspended solids. Colour coded reef categories (sensu Mellin et al. 2019) in panel b are: 1. Out-Soft - outershelf soft coral communities (dark blue), 2. Out-Digit - outershelf branching hard coral (light blue), 3. Out-Tab: outershelf tabular and corymbose hard coral (dark green), 4. Mid-Mixed - midshelf turf algae communities (light green), 5. In-Porites - inshore hard coral communities (orange), and 6. In-MA - inshore macroalgae communities (red). Water quality parameters differed strongly between inshore and outershelf reefs, characterised by lower dissolved organic (carbon, nitrogen and phosphorus) and inorganic (carbon and phosphorus) nutrients. The exceptions were inorganic nitrogen (DIN, mostly constituted by NO_3, but also NH_4) and chlorophyll *a* concentrations, with measured values peaking on midshelf reefs, particularly in the Out-Tab category.

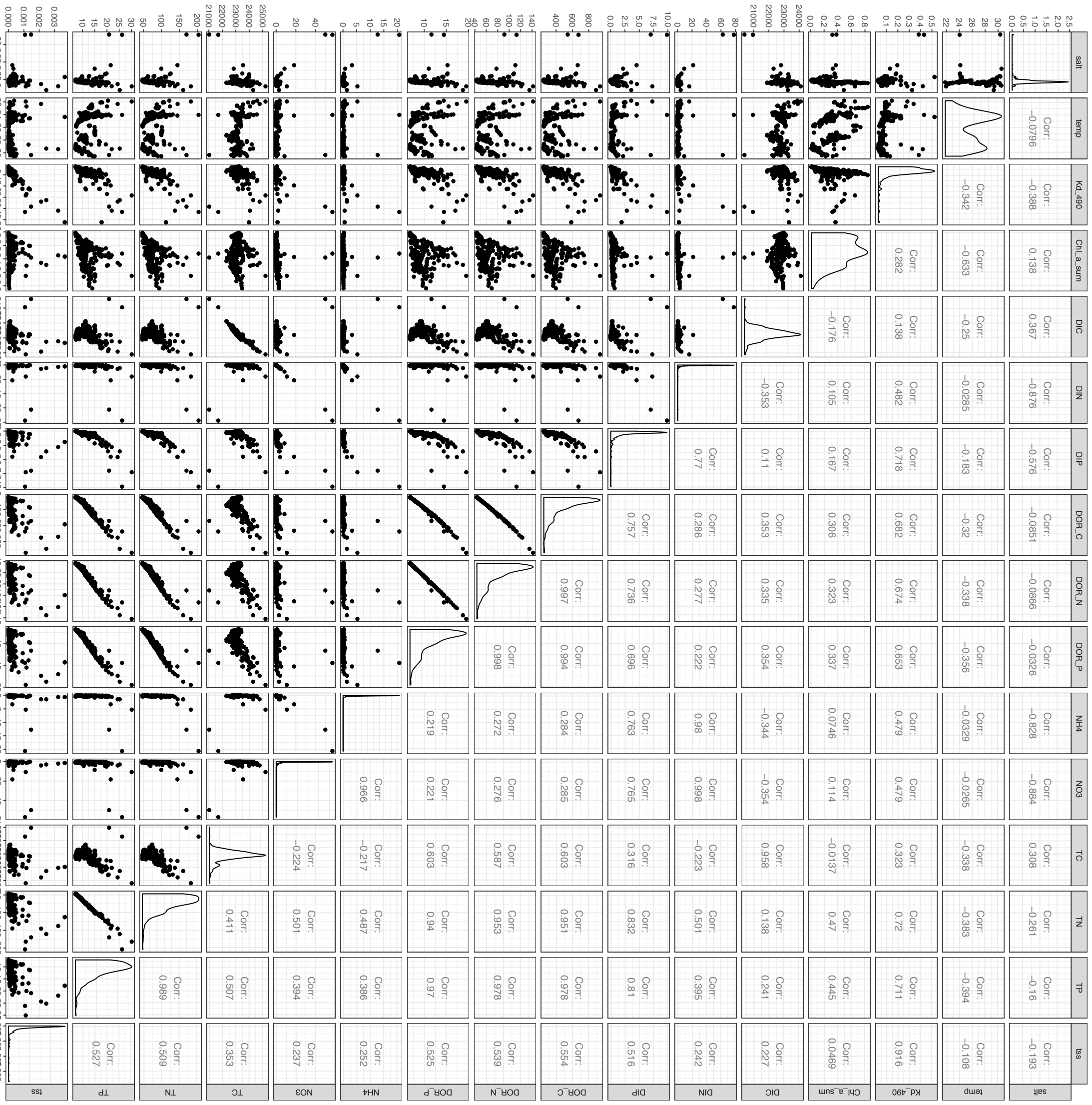

Supplementary Fig. 2. Correlations between pairs of variables to check for collinearity as basis for dimension reduction (based on all sites). Chl_a_sum: total chlorophyll *a*, DIC: dissolved inorganic carbon, DIN: dissolved inorganic nitrogen, DIP: dissolved inorganic phosphorus, DOR_C: dissolved organic carbon, DOR_N: dissolved organic nitrogen, DOR_P: dissolved organic phosphorus, KD_490: vertical attenuation coefficient of light at 490nm, NH4: ammonium, NO3: nitrate, salt: salinity, TC: total carbon, temp: temperature, TN: total nitrogen, TP: total phosphorus, and tss: total suspended solids.

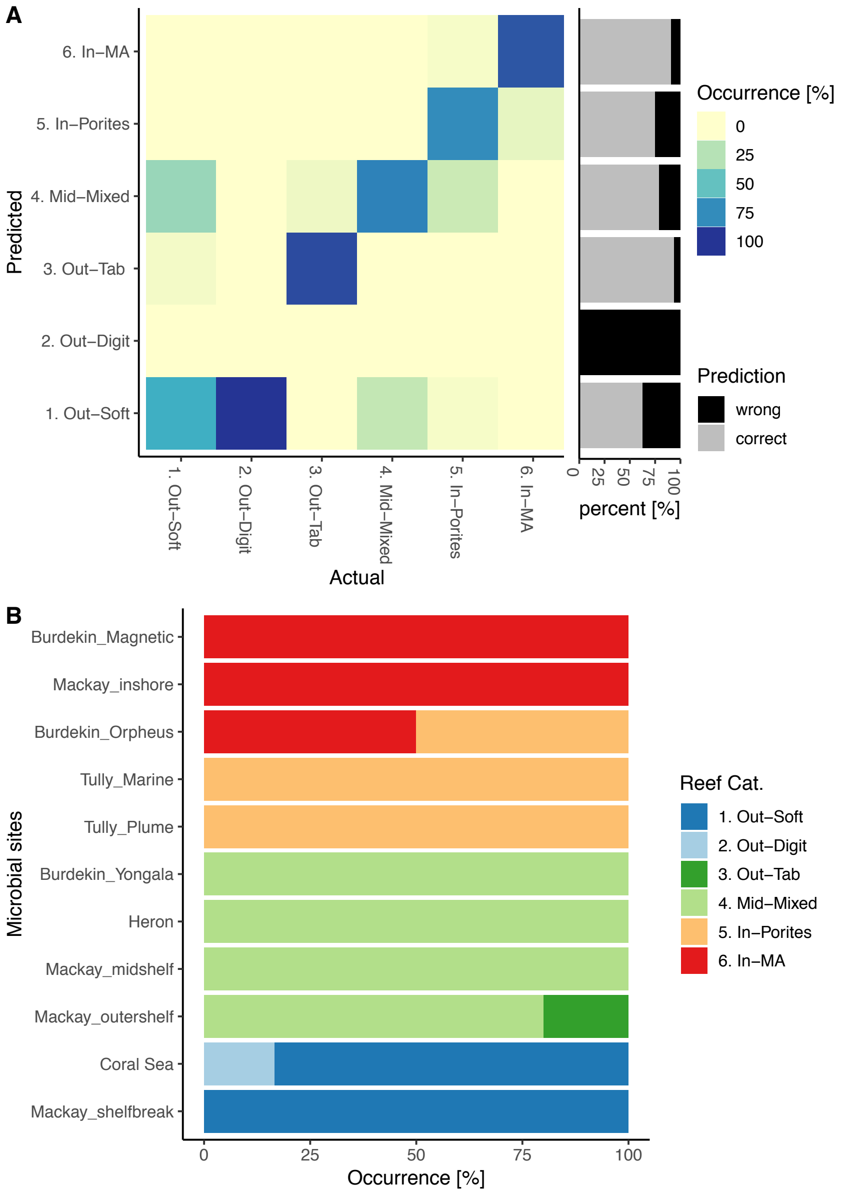

Supplementary Fig. 3. Assignment of linkage between microbial sites (case studies) and reef benthic categories using Linear Discriminant Analysis (LDA). a) Confusion matrix depicting actual (observed) vs predicted reef categories by the LDA model (total accuracy = 0.73 from a total of 109 test cases). Model accuracy was not constant across all reef categories. b) Scoring reef category affiliation of microbial sites following LDA modelling. Colour coded reef categories (sensu Mellin et al. 2019) in panel b are: 1. Out-Soft - outershelf soft coral communities (dark blue), 2. Out-Digit - outershelf branching hard coral (light blue), 3. Out-Tab: outershelf tabular and corymbose hard coral (dark green), 4. Mid-Mixed - midshelf turf algae communities (light green), 5. In-Porites - inshore hard coral communities (orange), and 6. In-MA - inshore macroalgae communities (red).

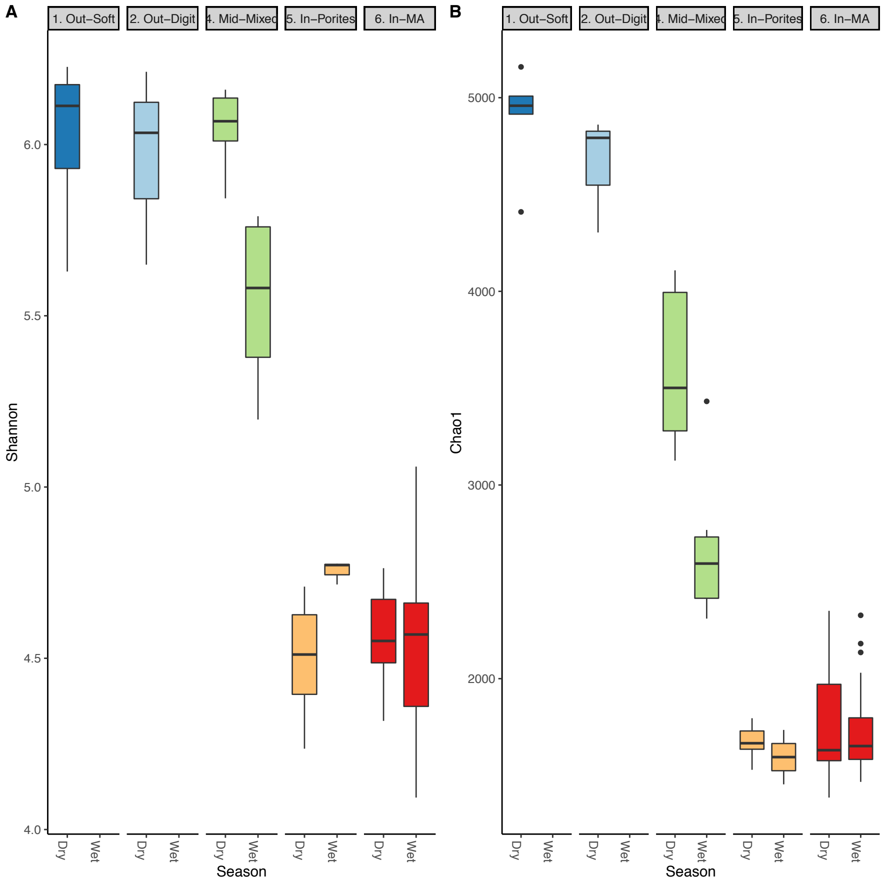

Supplementary Fig. 4. Alpha-diversity metrics across GBR reef categories. Shannon and Chao indexes. Colour coded reef categories (sensu Mellin et al. 2019) are: 1. Out-Soft - outershelf soft coral communities (dark blue), 2. Out-Digit - outershelf branching hard coral (light blue), 3. Out-Tab: outershelf tabular and corymbose hard coral (dark green), 4. Mid-Mixed - midshelf turf algae communities (light green), 5. In-Porites - inshore hard coral communities (orange), and 6. In-MA - inshore macroalgae communities (red).

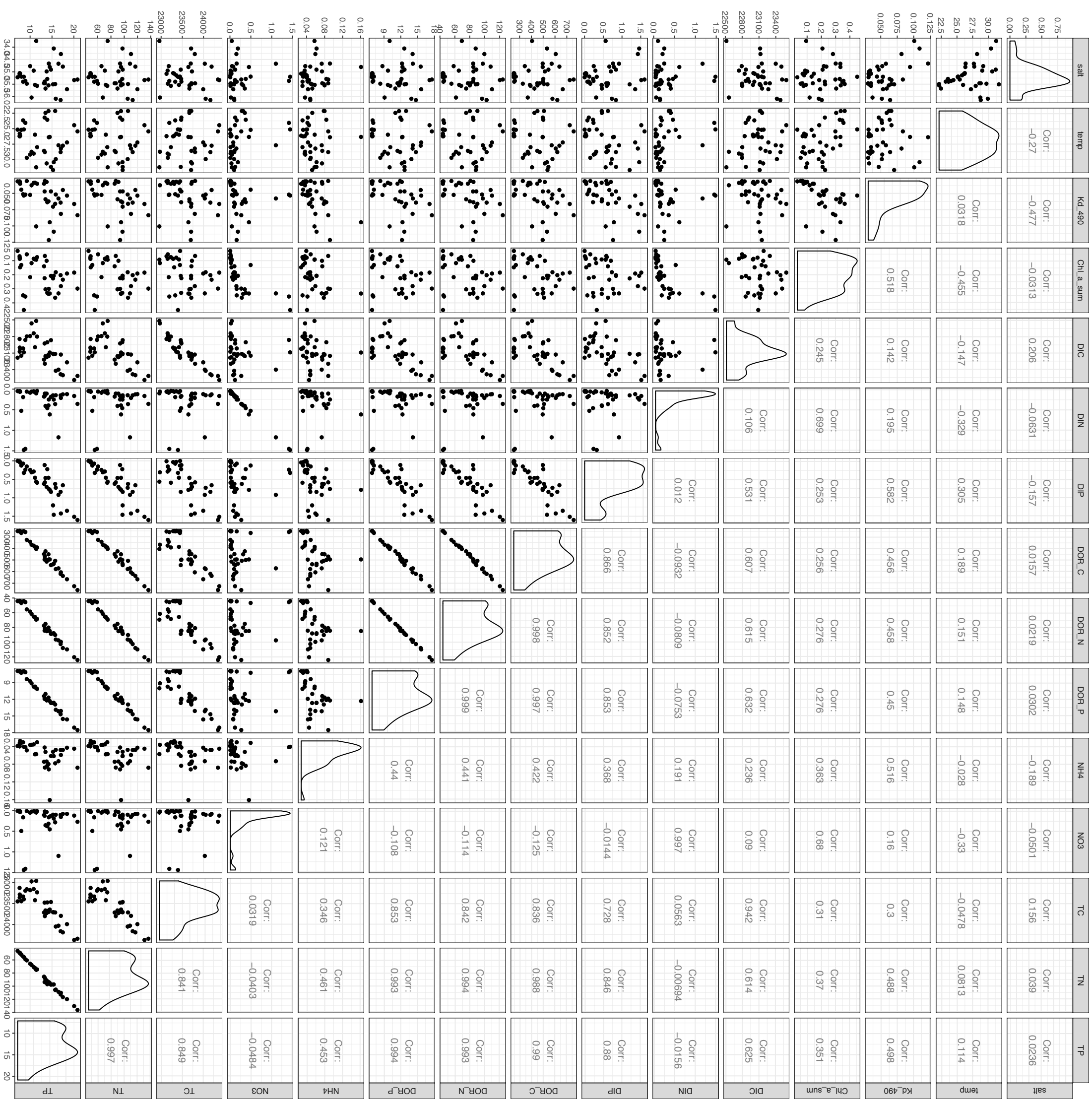

Supplementary Fig. 5. Correlations between pairs of variables to check for collinearity as basis for dimension reduction (based on microbial sites and 3d integration). Chl_a_sum: total chlorophyll *a*, DIC: dissolved inorganic carbon, DIN: dissolved inorganic nitrogen, DIP: dissolved inorganic phosphorus, DOR_C: dissolved organic carbon, DOR_N: dissolved organic nitrogen, DOR_P: dissolved organic phosphorus, KD_490: vertical attenuation coefficient of light at 490nm, NH4: ammonium, NO3: nitrate, salt: salinity, TC: total carbon, temp: temperature, TN: total nitrogen, TP: total phosphorus, and tss: total suspended solids.

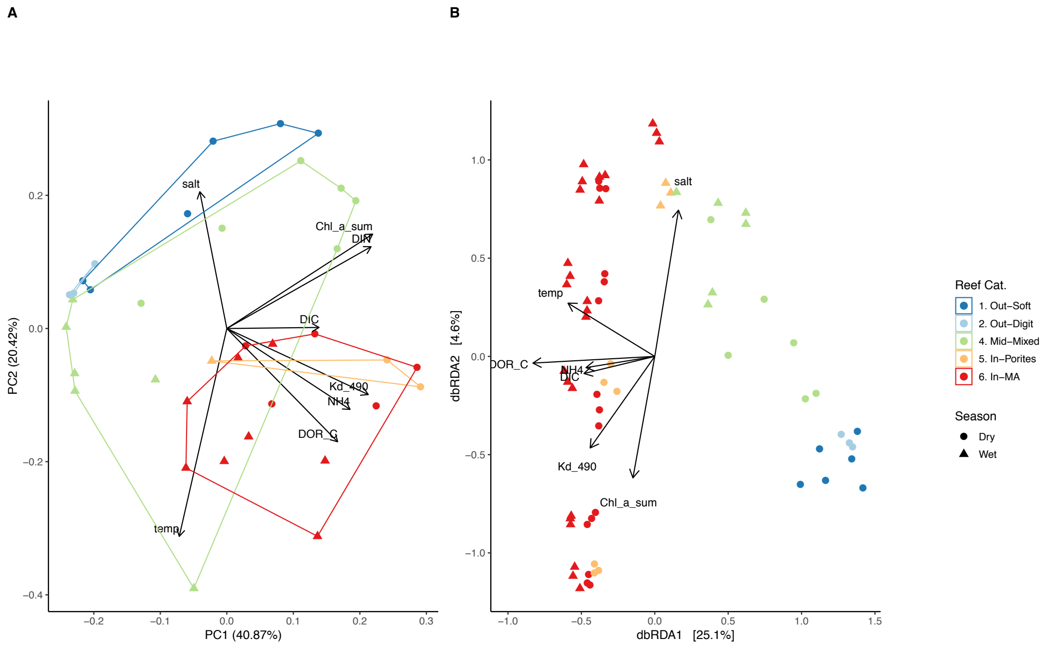

Supplementary Fig. 6. PCA-dbRDA for local microbial data and eReefs data. Chl_a_sum: total chlorophyll *a*, DIC: dissolved inorganic carbon, DIN: dissolved inorganic nitrogen, DOR_C: dissolved organic carbon, KD_490: vertical attenuation coefficient of light at 490nm, NH4: ammonium, salt: salinity and temp: temperature. Colour coded reef categories (sensu Mellin et al. 2019) are: 1. Out-Soft - outershelf soft coral communities (dark blue), 2. Out-Digit - outershelf branching hard coral (light blue), 3. Out-Tab: outershelf tabular and corymbose hard coral (dark green), 4. Mid-Mixed - midshelf turf algae communities (light green), 5. In-Porites - inshore hard coral communities (orange), and 6. In-MA - inshore macroalgae communities (red).

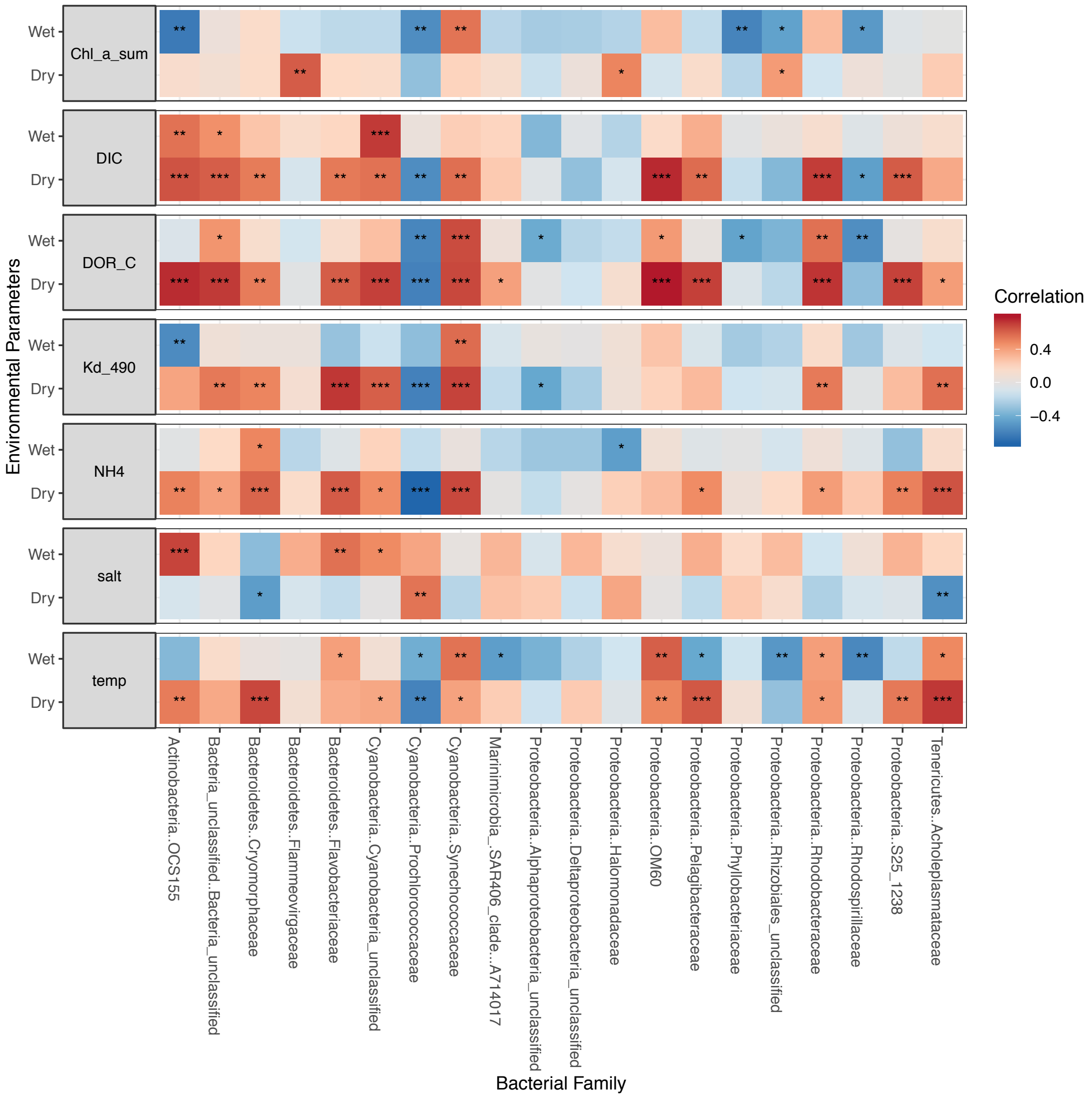

Supplementary Fig. 7. Correlations between significant environmental constraints of microbial community variation and the relative abundance of dominant individual bacterial families for the Great Barrier Reef (GBR). Chl_a_sum: total chlorophyll *a*, DIC: dissolved inorganic carbon, DOR_C: dissolved organic carbon, KD_490: vertical attenuation coefficient of light at 490nm, NH4: ammonium, salt: salinity and temp: temperature. Despite consistency of environmental-microbial correlations across seasons, for some microbial taxa-environmental parameter combinations there was a contrasting response between wet and dry seasons. For example, Pelagibacteraceae had a positive correlation with temperature in the cooler dry season but a negative one in the warmer wet season. This suggests Pelagibacteraceae has distinct and non-overlapping responses to temperature (note as well the non-overlapping temperature ranges between dry and wet season). Observing such correlations at seasonal resolution captures important trends that are otherwise obscured by the decoupling of seasonal and cross-shelf variation that happens for most environmental parameters; i.e. the effect of reef category is not stable over seasons. Again, seasonal

variation is more evident for inshore reefs than their offshore counterparts, where conditions seem to be more stable between summer (wet) and winter (dry).

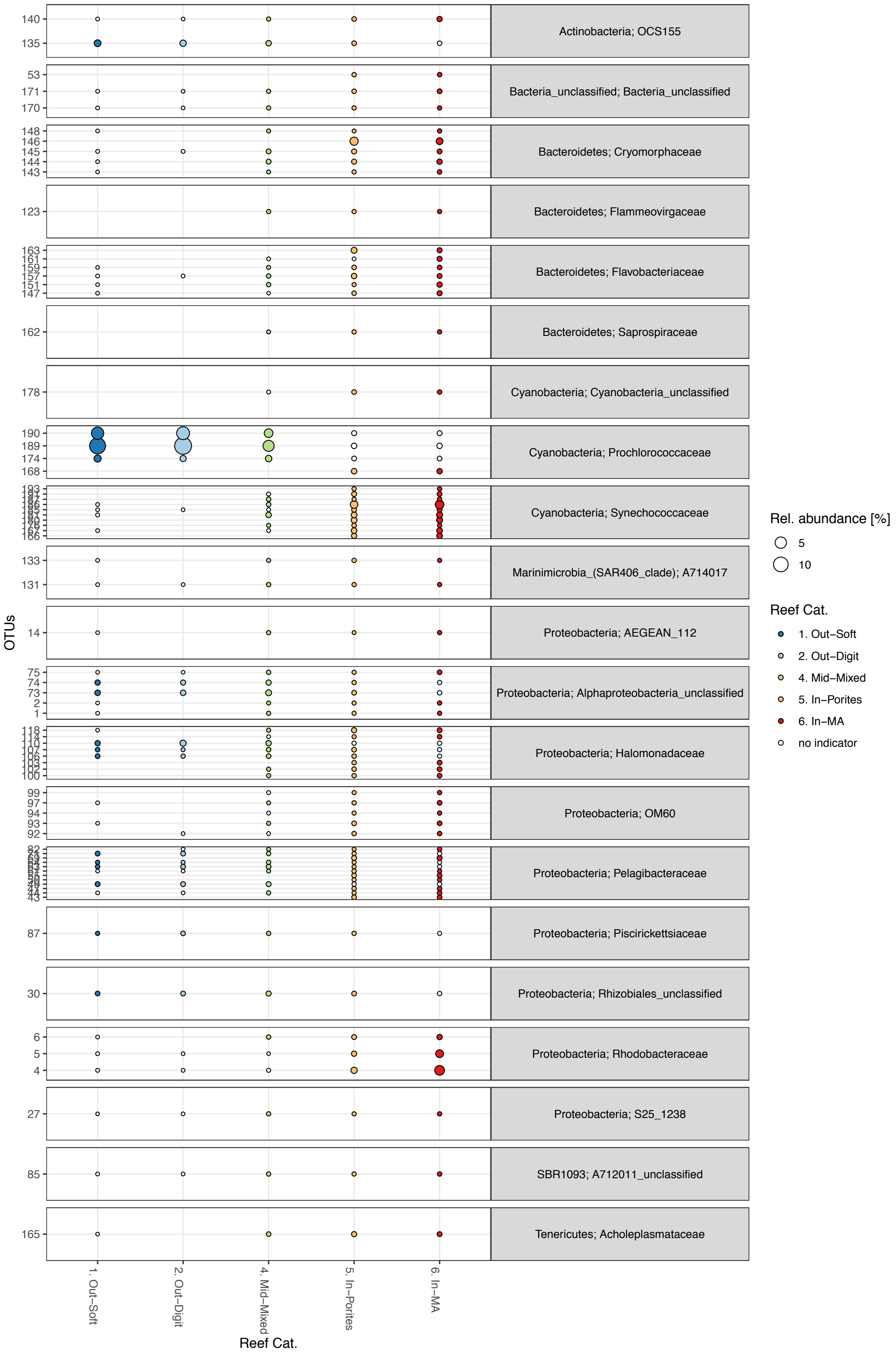

Supplementary Fig. 8. Individual indicator results for GBR benthic categories.

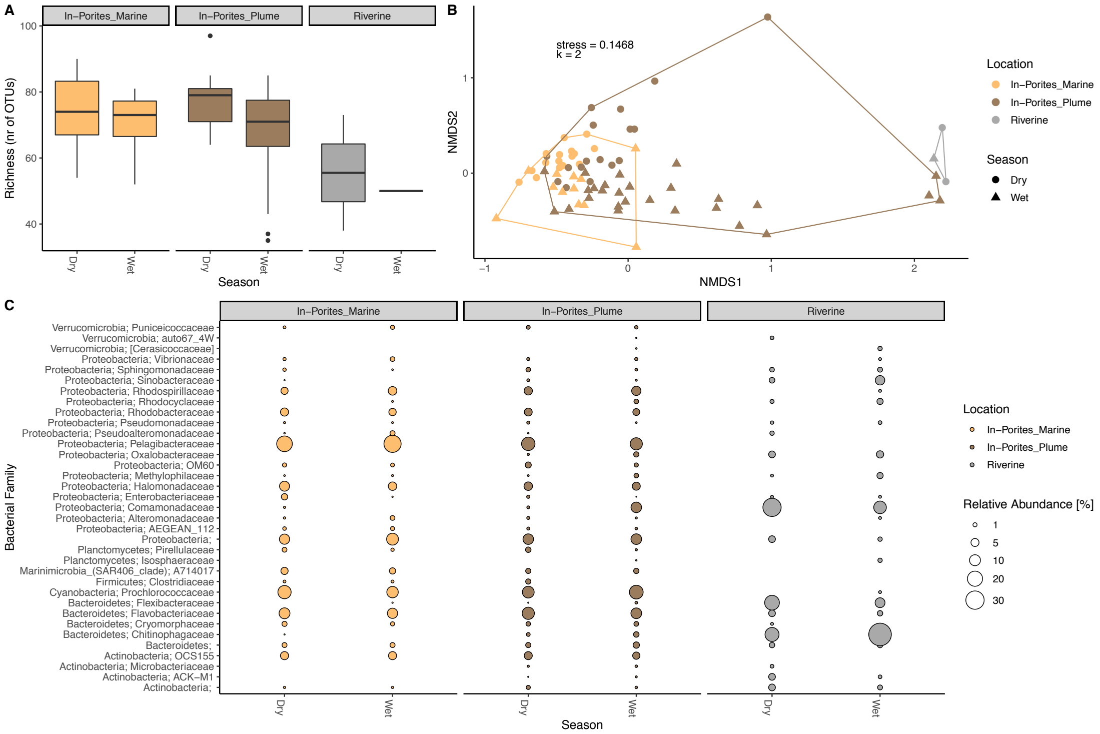

Supplementary Fig. 9. Microbial community descriptors for inter-seasonal plume influence across In-Porites reefs (Tully dataset; n=75). a) Alpha diversity (Richness, or observed OTU number per sample) across reef category and season (please see Supplementary Fig. 10 for other alpha-diversity indexes). b) Unconstrained ordination (nMDS) of In-Porites microbial communities based on plume influence and season. c) Microbial community composition of dominant bacterial families.

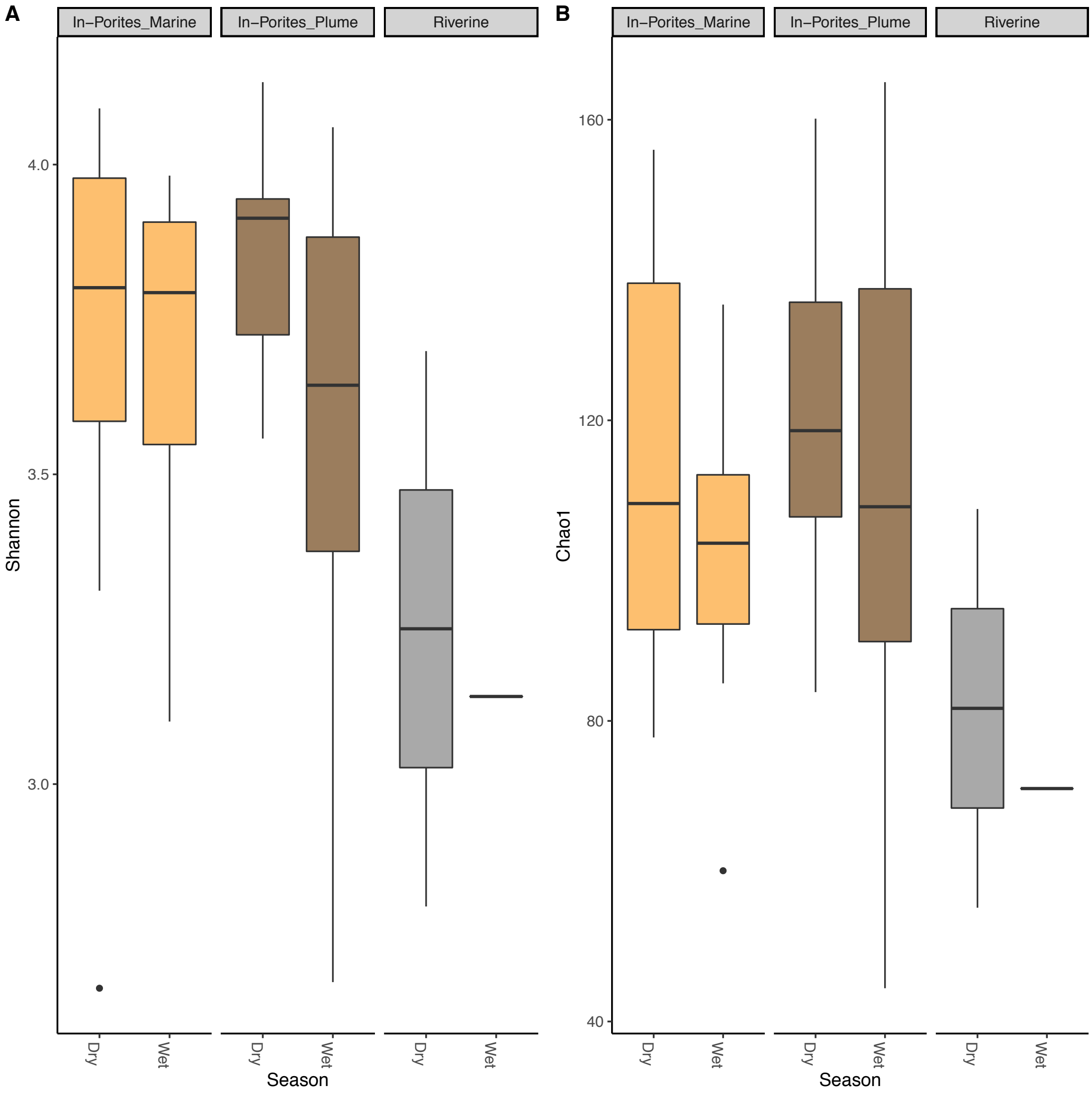

Supplementary Fig. 10. Alpha-diversity metrics across In-Porites seasons and locations (inside and outside river plume influence). Shannon and Chao indexes.

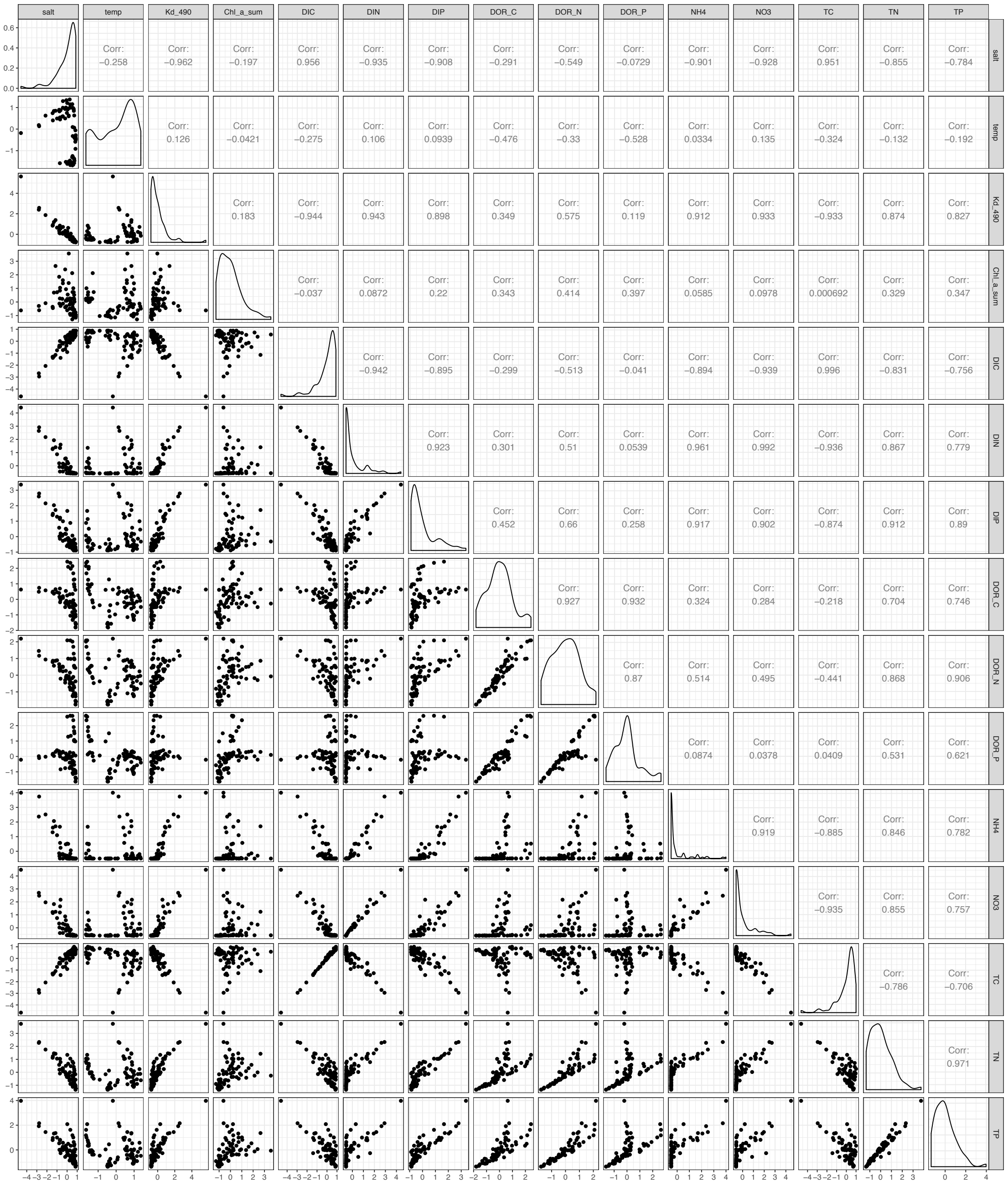

Supplementary Fig. 11. Correlations between pairs of variables for Tully dataset to check for collinearity as basis for dimension reduction (based on microbial sites and 3d integration). Chl_a_sum: total chlorophyll *a*, DIC: dissolved inorganic carbon, DIN: dissolved inorganic nitrogen, DIP: dissolved inorganic phosphorus, DOR_C: dissolved organic carbon, DOR_N: dissolved organic nitrogen, DOR_P: dissolved organic phosphorus, KD_490: vertical attenuation coefficient of light at 490nm, NH4: ammonium, NO3: nitrate, salt: salinity, TC: total

carbon, temp: temperature, TN: total nitrogen, TP: total phosphorus, and tss: total suspended solids.

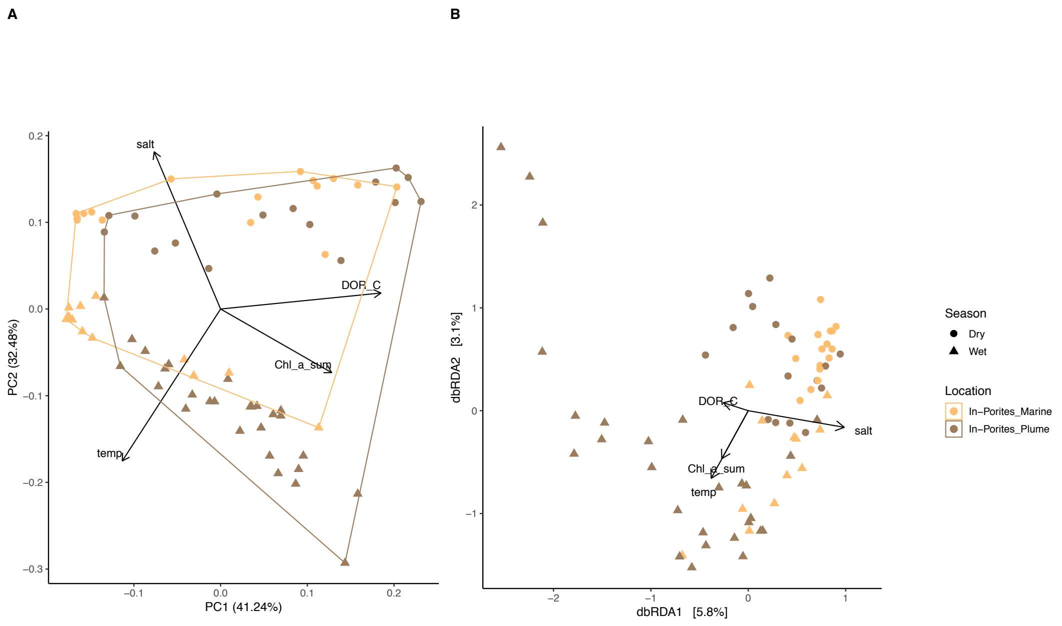

Supplementary Fig. 12. PCA-dbRDA for Tully microbial data and eReefs data. Chl_a_sum: total chlorophyll *a*, DOR_C: dissolved organic carbon, salt: salinity and temp: temperature. Note that n=3 Riverine samples were excluded due to absence of eReefs data for non-oceanic sites.

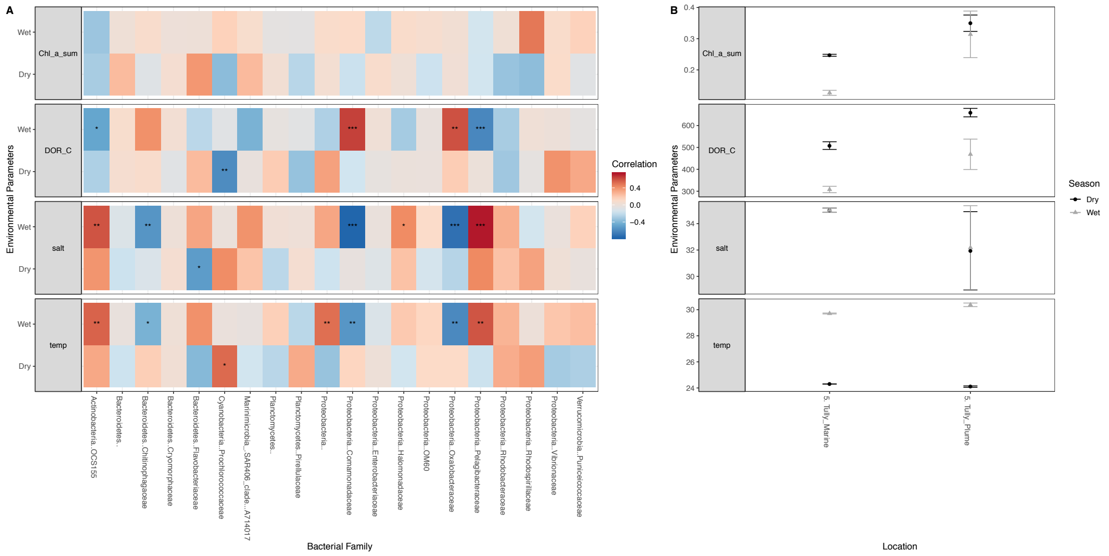

Supplementary Fig. 13. a) Correlations between significant environmental constraints of microbial community variation within the In-Porites reef category and the relative abundance of dominant individual bacterial families (Tully dataset; n=72). b) Environmental variation across the In-Porites plume categories for the main drivers of microbial community. Chl_a_sum: total chlorophyll *a*, DOR_C: dissolved organic carbon, salt: salinity and temp: temperature. Note that n=3 Riverine samples were excluded due to absence of eReefs data for non-oceanic sites.

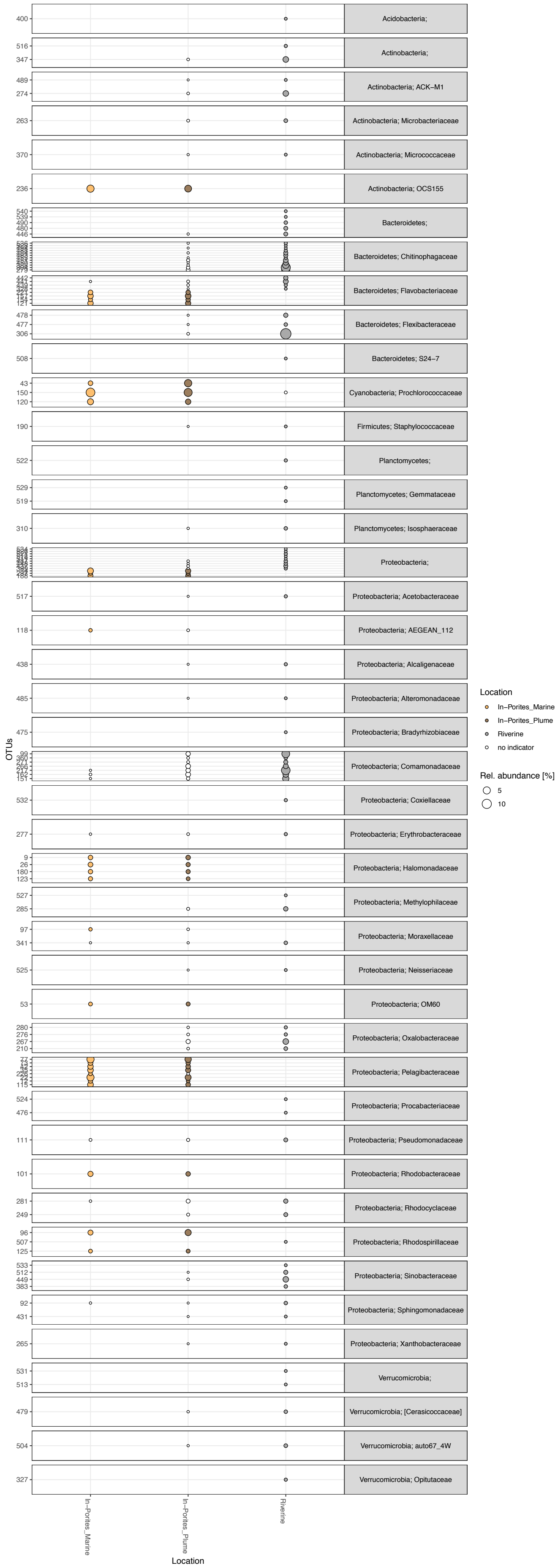

Supplementary Fig. 14. Individual indicator results for In-Porites plume categories (based on

Tully dataset; n=75).

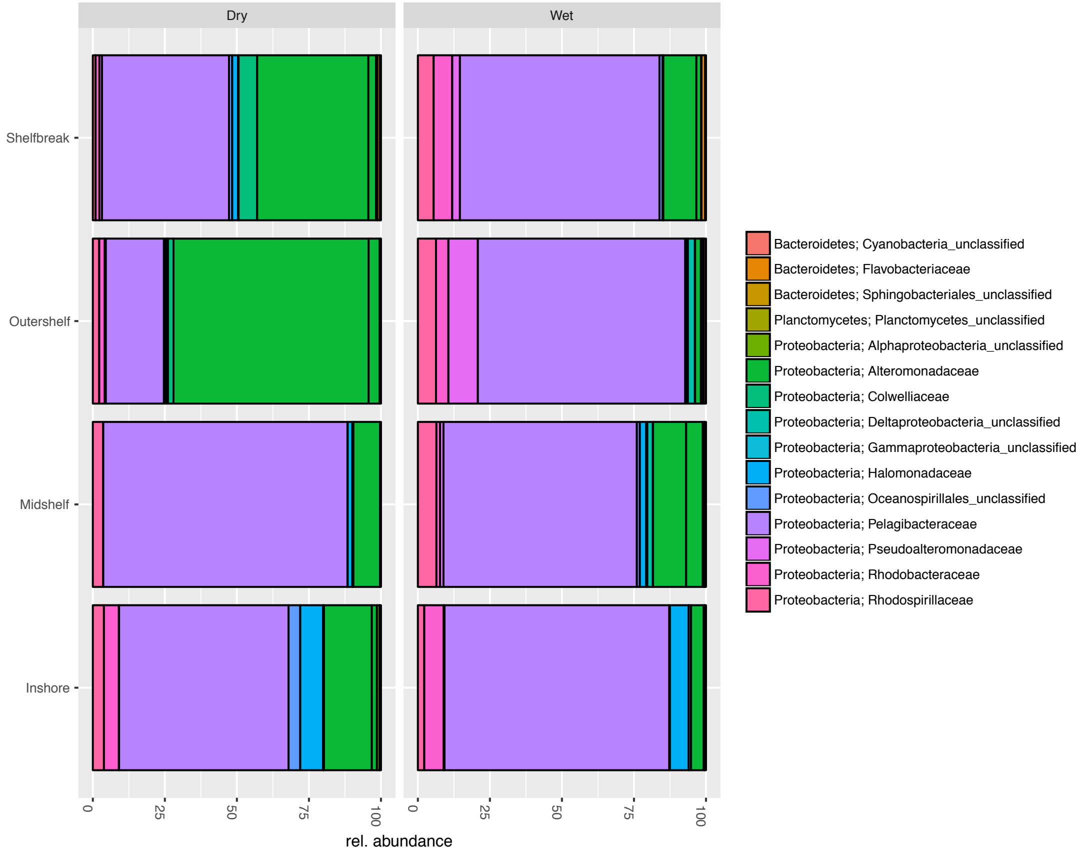

Supplementary Fig. 15. Family-level bacterial community composition for the Mackay region, with seasonal resolution. Each sampling group (or reef group) summarizes a number of locations. For simplicity, only the most abundant bacterial families across all samples are shown.

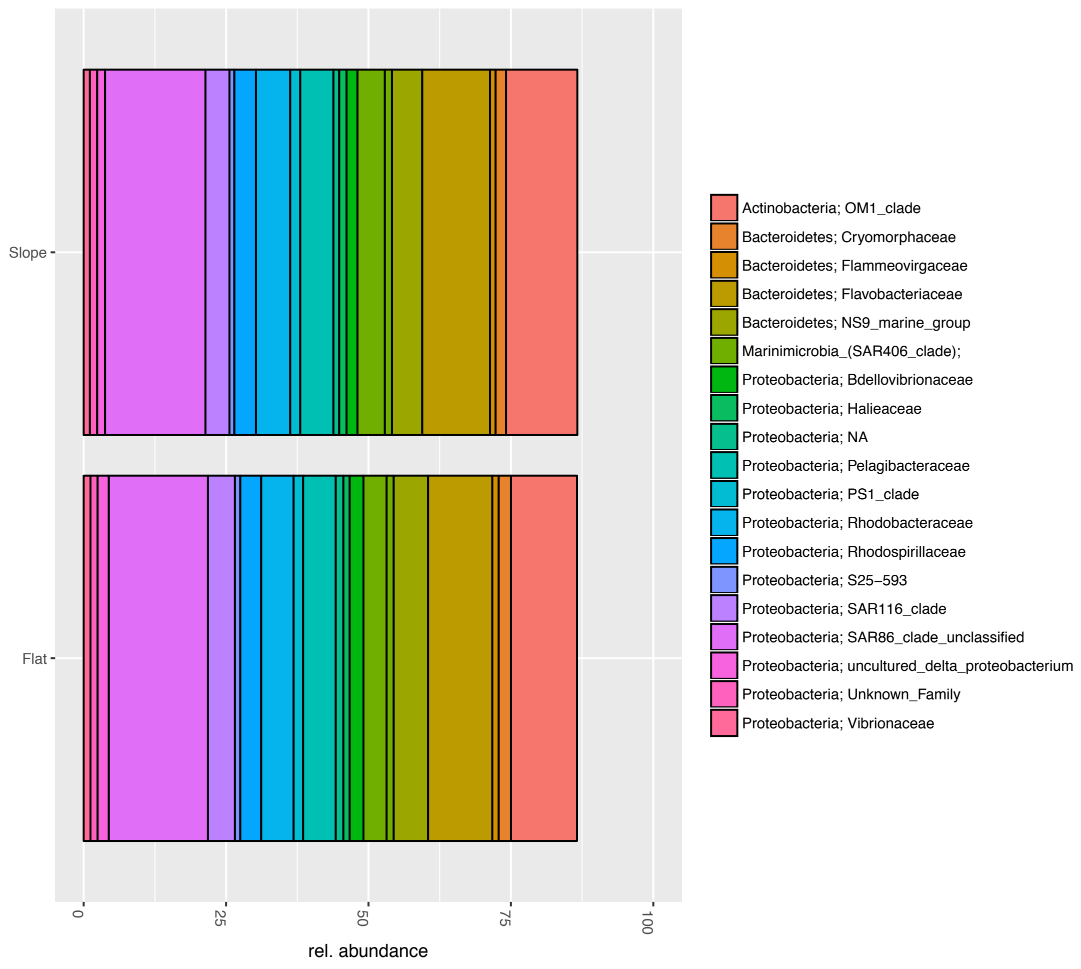

Supplementary Fig. 16. Family-level bacterial community composition for the Heron Island region, for the dry season only (no data available for the wet season). Each reef habitat consists of two different locations. For simplicity, only the most abundant bacterial families across all samples are shown.

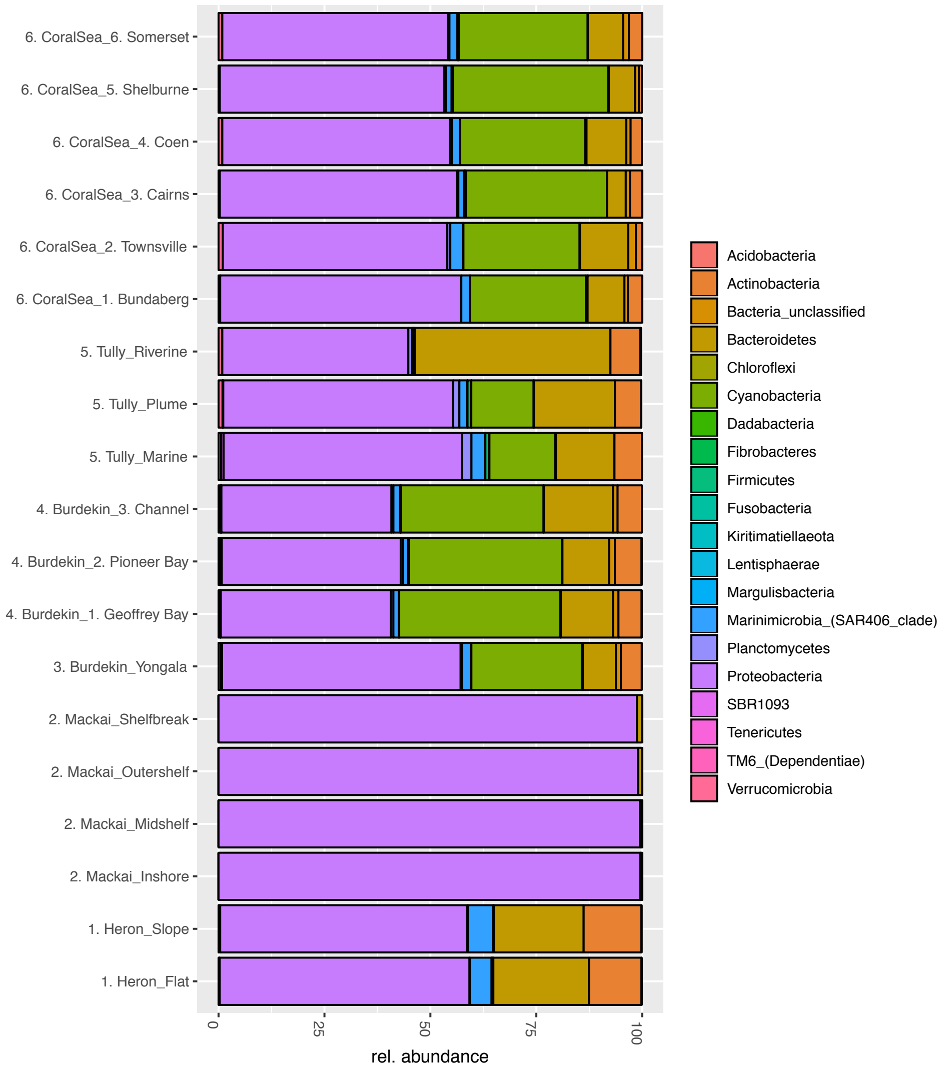

Supplementary Fig. 17A

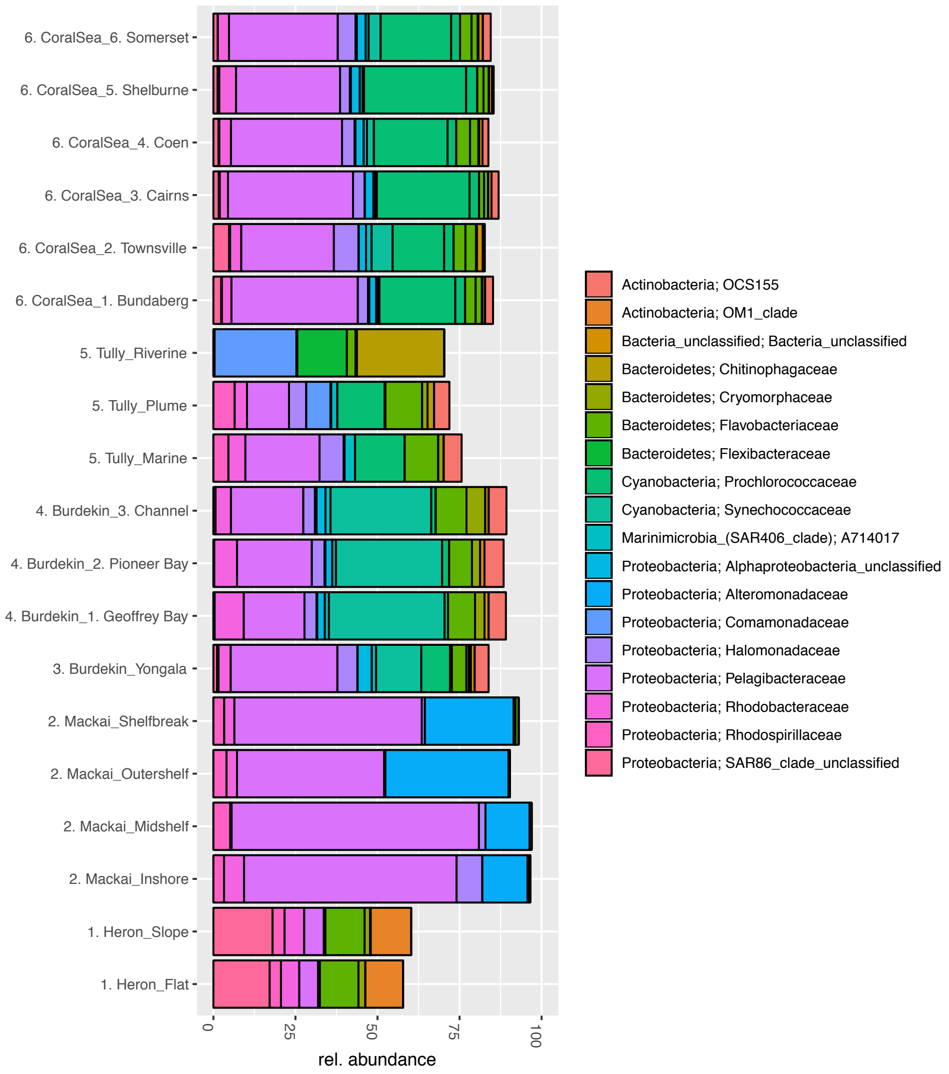

Supplementary Fig. 17B

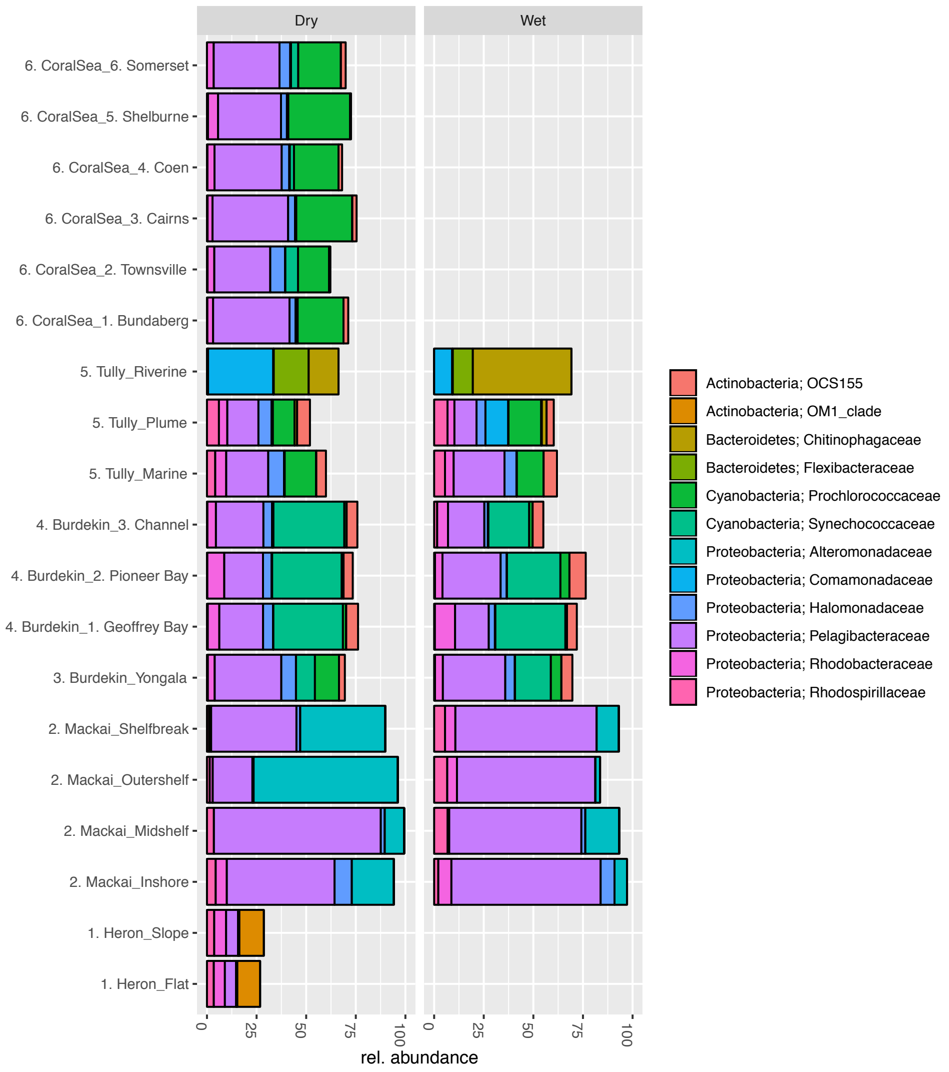

Supplementary Fig. 17C

Supplementary Fig. 17. Bacterial community composition across all regions, sampling groups and seasons obtained from the case-studies (n=147). a) Phylum-level and b) family-level community composition averaged across seasons, and c) with seasonal resolution. For simplicity, only the most abundant bacterial phyla across all samples are shown. Note that for this comparison data was rarefied to a depth of 1,000 reads and that 20 samples were consequently removed from the Tully dataset.

Supplementary Tables

Supplementary Table 1. Summary of published and unpublished microbial 16S rRNA datasets used in the meta-analysis, with respective study and region of origin, number of samples and locations included, primers used, and other methodological details. Note that the Glasl et al. (2019) dataset is also part of the BPA consortium (see Fig. 1).

Study	Region	Nr samples	Nr locations	Rarefaction depth	Sequencing platform	Taxonomic assignment	Primer pair and refs
Angly et al. 2016	Tully	75	7	250	454	SILVA and Greengenes	pyroLSSU926F/ pyroLSSU1392R
Glasl et al. 2019	Burdekin	48	3	25,000	Illumina Miseq 2x300	SILVA	27F/519R
BPA unpublished	Coral Sea	9	6	25,000	Illumina Miseq 2x300	SILVA	27F/519R
BPA unpublished	Yongala (Burdekin)	12	1	25,000	Illumina Miseq 2x300	SILVA	27F/519R
Epstein et al. 2019	Heron Island	15	4	50,000	Illumina Miseq 2x300	SILVA	515F/806Rb
Alongi et al. 2014	Mackay	8	4	1,350	454	GreenGenes	63F/533R

Supplementary Table 2. Alpha-diversity stats

Observed

```
Type III Analysis of Variance Table with Satterthwaite's method
                Sum Sq Mean Sq NumDF  DenDF F value    Pr(>F)    
Clust         10179736 2544934     4  2.000  82.685 0.0119853 *  
Season3         472996  472996     1 60.423  15.368 0.0002284 ***
Clust:Season3   888172  444086     2 60.527  14.428 7.518e-06 ***
---
Signif. codes:  0 ‘***’ 0.001 ‘**’ 0.01 ‘*’ 0.05 ‘.’ 0.1 ‘ ’ 1
```

```
ANOVA-like table for random-effects: Single term deletions
Model:
Observed ~ Clust + Season3 + (1 | Location) + Clust:Season3
               npar  logLik    AIC   LRT Df Pr(>Chisq)
<none>           10 -409.97 839.94                    
(1 | Location)    9 -410.47 838.94 1.005  1     0.3161
```

Shannon

```
Type III Analysis of Variance Table with Satterthwaite's method
              Sum Sq Mean Sq NumDF  DenDF F value    Pr(>F)    
Clust         9.9977 2.49943     4  2.000 59.5815 0.0165748 *  
Season3       0.0609 0.06092     1 60.558  1.4522 0.2328558    
Clust:Season3 0.8194 0.40972     2 60.630  9.7669 0.0002104 ***
---
Signif. codes:  0 ‘***’ 0.001 ‘**’ 0.01 ‘*’ 0.05 ‘.’ 0.1 ‘ ’ 1
```

ANOVA-like table for random-effects: Single term deletions

Model:

Shannon ~ Clust + Season3 + (1 | Location) + Clust:Season3

	npar	logLik	AIC	LRT	Df	Pr(>Chisq)
<none>	10	2.1246	15.751			
(1 | Location)	9	1.8947	14.210	0.4598	1	0.4977

Chao1

```
Type III Analysis of Variance Table with Satterthwaite's method
                Sum Sq Mean Sq NumDF  DenDF F value   Pr(>F)    
Clust         19477643 4869411     4  2.000  64.847 0.015244 *  
Season3        1050624 1050624     1 60.351  13.992 0.000411 ***
Clust:Season3  1939235  969617     2 60.456  12.913 2.14e-05 ***
---
Signif. codes:  0 ‘***’ 0.001 ‘**’ 0.01 ‘*’ 0.05 ‘.’ 0.1 ‘ ’ 1
```

ANOVA-like table for random-effects: Single term deletions

Model:

Chao1 ~ Clust + Season3 + (1 | Location) + Clust:Season3

	npar	logLik	AIC	LRT	Df	Pr(>Chisq)
<none>	10	-437.27	894.55			
(1 | Location)	9	-438.03	894.06	1.5128	1	0.2187

Supplementary Table 3. dbRDA stats - model selection (ordistep) and ANOVA to confirm significance of each constrain

```
                 R2.adj Df    AIC       F Pr(>F)
+ DOR_C         0.17046  1 152.92 14.9733  0.002 **
+ temp          0.24905  1 147.01  8.0121  0.002 **
+ salt          0.27992  1 145.07  3.8289  0.002 **
+ NH4           0.30520  1 143.53  3.3655  0.002 **
+ DIC           0.32234  1 142.72  2.6181  0.002 **
+ Chl_a_sum     0.33588  1 142.22  2.2847  0.002 **
+ Kd_490           1 142.01  1.9916  0.006 **
<All variables> 0.35090
---
Signif. codes:  0 ‘***’ 0.001 ‘**’ 0.01 ‘*’ 0.05 ‘.’ 0.1 ‘ ’ 1
```

```
Permutation test for dbrda under direct model
Terms added sequentially (first to last)
Permutation: free
Number of permutations: 9999

Model: dbrda(formula = matrix ~ DOR_C + temp + salt + NH4 + DIC +
Chl_a_sum + Kd_490, data = abiotic, distance = "bray", sqrt.dist
= TRUE, na.action = na.omit)
          Df SumOfSqs       F Pr(>F)
DOR_C      1   1.9629 19.0020 0.0001 ***
temp       1   0.9508  9.2045 0.0001 ***
salt       1   0.4357  4.2179 0.0004 ***
NH4        1   0.3695  3.5773 0.0011 **
DIC        1   0.2804  2.7143 0.0064 **
Chl_a_sum  1   0.2398  2.3213 0.0145 *
Kd_490     1   0.2057  1.9916 0.0304 *
Residual  61   6.3014
---
Signif. codes:  0 ‘***’ 0.001 ‘**’ 0.01 ‘*’ 0.05 ‘.’ 0.1 ‘ ’ 1
```

Supplementary Table 4. Alpha-diversity stats for Tully dataset. Note that Riverine location was excluded from analysis as only n=3 samples were available.

Observed

	Df	Sum Sq	Mean Sq	F value	Pr(>F)	
Season3	1	959	959.4	7.935	0.00634	**
location	1	1	0.7	0.006	0.93826	
Season3:location	1	99	98.7	0.816	0.36949	
Residuals	68	8222	120.9			

Shannon

	Df	Sum Sq	Mean Sq	F value	Pr(>F)	
Season3	1	0.593	0.5935	5.793	0.0188	*
location	1	0.005	0.0046	0.045	0.8324	
Season3:location	1	0.256	0.2559	2.498	0.1186	
Residuals	68	6.966	0.1024			

Chao1

	Df	Sum Sq	Mean Sq	F value	Pr(>F)
Season3	1	1585	1584.8	2.146	0.148
location	1	477	476.9	0.646	0.424
Season3:location	1	0	0.0	0.000	0.999
Residuals	68	50208	738.4		

Supplementary Table 5. dbRDA stats - model selection (ordistep) and ANOVA to confirm significance of each constrain for Tully dataset

```
                  R2.adj Df    AIC      F Pr(>F)
+ salt          0.043517  1 233.21 4.1848  0.002 **
+ temp          0.055010  1 233.32 1.8392  0.002 **
+ Chl_a_sum     0.063497  1 233.62 1.6162  0.002 **
+ DOR_C         0.067156  1 234.28 1.2628  0.008 **
<All variables> 0.067156
---

Permutation test for dbrda under direct model
Terms added sequentially (first to last)
Permutation: free
Number of permutations: 9999

Model: dbrda(formula = matrix ~ salt + temp + Chl_a_sum + DOR_C,
data = abiotic, distance = "bray", sqrt.dist = TRUE, na.action =
na.omit)
          Df SumOfSqs      F Pr(>F)
salt       1   1.5413 4.2673 0.0001 ***
temp       1   0.6746 1.8678 0.0003 ***
Chl_a_sum  1   0.5893 1.6315 0.0014 **
DOR_C      1   0.4567 1.2644 0.0283 *
Residual  66  23.8391
---
```

Supplementary Results & Discussion

Detailed results on the environmental variation across GBR surface waters

Modelled estimates of the environmental conditions of surface seawater retrieved from the eReefs hydrodynamic and biogeochemical model (GBR1, https://research.csiro.au/ereefs/models/model-outputs/gbr1/) for n=37 microbial case study sites and n=109 LTMP sites (see Fig. 1) covered 16 different environmental variables: salinity, temperature, total chlorophyll *a*, dissolved inorganic carbon (DIC), nitrogen (DIN) and phosphorus (DIP), ammonium (NH4), nitrate (NO3), dissolved organic carbon (DOC), nitrogen (DON) and phosphorus (DOP), total carbon (TC), nitrogen (TN) and phosphorus (TP), total suspended solids (TSS) and the vertical attenuation coefficient of light (Kd_490). Overall, organic and inorganic nutrients decreased in concentration with increasing distance from the shore (Supplementary Fig. 1; In-MA and In-Porites > Mid-Mixed and Out-Tab > Out-Soft and Out-Digit). The exception was the inorganic nitrogen variables (DIN, NH4 and NO3), which, together with chlorophyll *a*, peaked at midshelf reefs, particularly in the Out-Tab reef category. Superimposed on this inshore to outershelf trend, there was strong seasonal variation. However, this effect tended to lose its influence towards outershelf categories (Out-Soft and Out-Digit). Out-Soft and Out-Digit were devoid of seasonal effects, with the exception of temperature differences between the wet and dry seasons. Inshore reef categories in contrast, showed strong seasonal differences for all variables measured, and nutrient concentrations reached higher values during the dry season than the wet season.

Environmental variation was modelled by LDA to predict reef category for the sites with available microbial community data. Salinity, temperature, chlorophyll *a*, Kd_490, DOC and DIC were non-collinear variables (see Supplementary Fig. 2) and were thus included in the LDA modelling (Supplementary Fig. 3).

Bacterial response to riverine and seasonal influences within inshore In-Porites reefs

In contrast to the BPA dataset used to compare microbiomes across GBR reef categories, the Tully dataset[1] included only sites assigned by our LDA model to the In-Porites reef category. The importance of the Tully microbiome dataset is that it encompasses both the spatial dynamics generated by the outflow of the Tully river onto the inshore reef, as well as the superimposed temporal dynamics established between dry and wet seasons, and can be used to understand microbial variation within a single GBR reef category.

Alpha diversity (Richness; Supplementary Fig. 9a) varied significantly with season but not with location (no effect of plume versus marine locations) (see Supplementary Table 4 and Suppl. Fig. 10 for further results). Microbial diversity was higher in the dry season than in the

wet season, what is consistent with the BPA dataset. Riverine sites showed a much lower diversity than the plume and marine sites, which did not differ from one another. nMDS (Supplementary Fig. 9b) shows some overlap between communities based on location, but a clearer structuration of the community based on season, particularly for the plume sites. Season differences and general heterogeneity seems to be more marked for the plume sites, whereas samples belonging to the marine sites are more similar among each other. PERMANOVA showed that there was a location and season effect on microbiome structure, however no interaction (full model PERMANOVA, pseudo $F_{(69, 2)} = 3.6719$, $p = 0.0001$).

Shallow-water pelagic microbiomes in the In-Porites reef category of the Tully region are dominated by Pelagibacteraceae, Prochlorococcaceae and Flavobacteracea (Supplementary Fig. 9c). This dataset displayed cyanobacterial dominance by the Prochlorococcaceae family, as opposed to Synechococcaceae that dominated the BPA dataset. Moreover, in the Tully dataset there was an overall reduction in Cyanobacteria and an increase in the relative abundance of Rhodospirillaceae. It is likely that dominance of Prochlorococcaceae is attributed to primer bias (different primer sets were used between studies) that excluded amplification of Synechococcaceae. This exemplifies the need for caution when making comparisons across datasets obtained with different methods. Locations under the influence of the river plume display a microbial community characterized by an abundance of the bacterial taxa Bacteroidetes, Chitinophagaceae, Comamonadaceae, Proteobacteria and Oxalobacteraceae (see Supplementary Fig. 9). Further offshore and away from the mouth of the Tully River, microbial communities are consistent with inshore Burdekin sites, dominated by Pelagibacteraceae and Cyanobacteria, Flavobacteraceae (Bacteroidetes), Rhodobacteraceae (Proteobacteria), family OCS155 (Actinobacteria), Cryomorphaceae (Bacteroidetes), Halomonadaceae (Proteobacteria), the poorly described SAR406, and also Rhodospirillaceae (Proteobacteria). This profile is typical of pelagic microbial communities throughout the inshore reef locations studied.

Environmental variation in the Tully region (4x4 Km eReefs data) was mostly explained by four environmental parameters (DOC, salinity, temperature and chla) after dimension reduction via subtraction of collinear variables (from 16 original variables; Supplementary Fig. 11). The first two components of PCA represented 73.7% of variation in the dataset with n=73 samples included; Supplementary Fig. 12). dbRDA (Supplementary Fig. 12) proceeded with n=71 microbial samples and model selection showed that all of the four constrains used explained significant variation in the microbial community (see Supplementary Table 5). Full model (no interactions considered) was significant according to an ANOVA-like permutational test (pseudo $F_{(66,4)} = 2.2598$, $p < 0.01$). Total explanatory value of significant constrains was 12.0%, according to Variation Partitioning Analysis. Percentage of variation explained by individual constrains was 5.7% for salinity, 2.9% for temperature, 2.3% for chlorophyll *a*, and

1.8% for DOC. The very low percentage of variation explained by significant constrains (12.0%) suggests that there is a lot of local variation beyond the one explained by the measured environmental variables. Regardless, microbial differences (see Supplementary Fig. 12) are concurrent with both a higher DOC (as well as collinear DON and DOP) and lower salinity (and lower DIC, but higher DIN, DIP, NH4 and NO3, and Kd) in the plume sites than in the more oceanic sites (marine sites). In parallel, temperature and chlorophyll levels were higher in the wet season than in the dry season, and constitute the other significant drivers of microbial variation.

Correlations between individual environmental parameters and individual bacterial families across In-Porites sites with different plume influence (see Supplementary Fig. 13) were not as explicit as the ones found across different GBR reef categories. Nevertheless, increasing organic nutrient loads (DOC, as well as DON and DOP) and decreasing salinity (and lower DIC, but higher DIN, DIP, NH4 and NO3, and Kd), positively correlate with an increase in families Oxalobacteraceae and Comamonadaceae during the wet season, and with a decrease in Pelagibacteraceae and OC155 in the wet season. Furthermore, increased DOC correlated with decreased Prochlorococcaceae in the dry season, and increased salinity with an increase in Halomonadaceae and a decrease in Chitinofagaceae in the wet season, and a decrease in Flavobacteraceae in the dry season. The effects of increasing temperature were very similar to those of increasing salinity. Higher temperature correlated with a higher relative abundance of Pelagibacteraceae and OC155 in the wet season, and of Prochlorococcaceae in the dry season, but with a lower relative abundance of Oxalobacteraceae, Comamonadaceae and Chitinophagaceae during the wet season. Chlorophyll, the other significant driver of microbial community, did not significant correlate with any of the most abundant bacterial families.

Perhaps not surprisingly, our indicator value analysis found no single indicator exclusive for the plume locations of In-Porites reefs (Supplementary Fig. 14). All indicators of the plume environment were either shared with the marine or the riverine locations, again demonstrating the transient nature of the plume water body. On the other hand, there were several indicators of the riverine system, but just a few for the marine locations.

Hence, proximity to river mouths can drive microbial community dynamics which increase taxa in the surrounding waters that have been implicated as causing corals diseases[2]. Marine Group II Euryarchaeota were also more abundant during the wet season[1]. These motile residents of the photic zone that have a photo-heterotrophic lifestyle through which they degrade protein and lipids, are also known to display great seasonal and spatial variation elsewhere[3,4]. Most likely there are also important numbers of Archaea across the abovementioned inshore to offshore gradient, as well as within each of the regions here

characterized. Archaea have not been characterized widely (see, for example, Frade et al.[5]) and their importance for coral reef functioning is far from being understood.

It is noteworthy that reefs in the Tully region show coral cover (46-51%) similar to that of Orpheus Island and higher than that of Magnetic Island, showing that the region seems to be in a good conservation state even after being hit by recent cyclones[6]. Even locations under the effect of a river plume can still sustain reasonable coral (11-32%) and low macroalgal cover (2-8%).

Shelf edge effects on bacterial community profiles.

The dataset derived from the Mackay region[7], allows investigation of the influence of coastal distance and reef shelf on microbial community structure (Fig. 1). From the inshore and midshelf to the outershelf and shelfbreak there is large increase in relative abundance of Alteromonadaceae and a concomitant decrease in Pelagibacteraceae and the Halomonadaceae (Supplementary Fig. 15). Major differences in community composition between the different locations studied across the GBR shelf occur during the dry season. Whereas the seasonal effects for Tully region are likely related to terrestrial run-off and riverine incursion into marine communities along the inshore reefs[1,6], seasonal drivers in the Mackay region could be of oceanic origin. The region is highly productive due to upwelling along the shelf, bringing nutrients and cold water from the deep and therefore contributing to shaping the microbial communities of outer reefs. However, the upwelling regime in that area is restricted to the summer wet months[8], but the nutrient data available does not support a hypothesis of upwelling as a driver of changing microbial community structure[7]. This may be related to the very restricted temporal window during which samples were taken in the region. One important characteristic of the GBR in this region is the extensive distance of the outer reef from shore. This and the prevalent upwelling system that is active during the wet season could contribute to differences seen in community composition. Alteromonadaceae are recognized as copiotrophs that can grow rapidly when organic nutrients are available in the environment[9,10].

Bacterial community patterns in the southern GBR

The final data set is derived from Heron Island and presents a different microbial community compared to the more northern regions (see Supplementary Fig. 16). Methodological biases likely contributed to these differences, which are reflected mainly by an absence of Cyanobacteria. This site however is dominated by similar groups identified for the Burdekin lagoon and Coral Sea regions. For example, Pelagibacteraceae, SAR86 (now very dominant), SAR406, Rhodobacteraceae and Rhodospirillaceae, and Cryomorphaceae are abundant taxa. Other dominant groups include the SAR116, the Bacteroidetes NS9 and Flavobacteraceae, as well as OM1 clade in the Actinobacteria. It is interesting to note that

community assemblages are almost identical between the reef flat and reef slope (2 and 6 m depth, respectively) at this site. The increase in the proteobacterial clades SAR86 and SAR116 as well in OM1 clade (Actinobacteria), all known for their streamlined genomes[11], likely reflect the oligotrophic conditions in the system. However dominance of Flavobacteraceae, may suggest an increased abundance of opportunistic bacteria that may affect coral health[10,12].

Methodological considerations

In order to incorporate available microbial community data from current case studies on the GBR with the reef categorization of Mellin et al.[13] and to predict microbial community composition across the wider GBR, we started by comparing the prevailing environmental conditions found at the case study locations with those found for n=109 sites previously assigned to reef categories. This comparative framework identified with an overall model accuracy of 73.4%, the reef category affiliation of each microbial case study location. Out of six recognized reef benthic categories identified by Mellin et al.[13], all except Out-Digit were recovered with an accuracy above 62%, and with predictive values as high as 90.9% for In-MA, 75.0% for In-Porites, 78.9% for Mid-Mixed, 93.8% for Out-Tab and 62.5 for Out-Soft (see percent correct predictions in Supplementary Fig. 3a). Out-Digit coincides with poor model recovery of this category (0% accuracy according to cross validation). There was a tendency for the LDA model to bleed observations in the Out-Soft category into Mid-Mixed predictions, and vice-versa. The poor model recovery of Out-Digit and substantial error rates in assignments of Out-Soft (37.5%) and Mid-Mixed (21.1%) are likely a consequence of the increasing overlap of environmental conditions in reef categories representing outershelf and midshelf reefs as compared to the more discrete and divergent conditions of inshore categories (see Supplementary Fig. 1). Coastal inshore reefs are under the influence of heterogeneous terrestrial inputs over both space and time[14], in addition to strong seasonal atmospheric and terrestrial fluctuations[15]. This is in contrast to reefs further offshore with conditions of Out-Digit and Out-Soft reefs similar across seasons (Supplementary Fig. 1). The original reef classification scheme developed by Mellin et al.[13] incorporated the seasonal range of seabed temperature, a parameter not included in our analyses since the microbiome community data available is derived predominantly from surface waters. In contrast, the two inshore categories, In-MA and In-Porites, segregated according to the seasonal range of sea surface temperature[13] and their recovery by our LDA model was therefore efficient. For n=29 microbial sites that happen to be also included in the GBR wide predictions of Mellin et al.[13], we found a 72.4% match in inferred reef category between our LDA model and their multivariate regression tree. This was confirmed by a Cohen's weighted Kappa of 94.2%. Incongruent predictions mostly corresponded to "Out-Soft - Out-Digit" and "Out-Tab - Mid-Mixed" mismatches. These again correspond to the space where our LDA model has the lowest resolution to tease apart reef categories. Reduced model performance for the

outershelf reefs hinders our capacity to clearly identify differences in microbial communities correlated with Out-Digit and Out-Soft. In addition, none of the locations providing microbial data overlapped with Out-Tab with respect to environmental variation (modelled by LDA), thus we could not predict microbial community composition for this reef category.

Caveats of this meta-analysis

The datasets originating from the Burdekin region, including the Yongala lagoon site, plus the Coral Sea dataset were obtained with the same primer set and processed through the same analysis pipeline of BPA[16,17]. This allowed for a fairly robust comparison across sites and distinct reef categories. For comparisons across all available datasets, reads were further rarefied to a depth of 1,000 reads (See Supplementary Fig. 17 for a comparative perspective across all datasets). The very low abundance of Cyanobacteria in the Heron Island dataset, and the unaccompanied dominance of Proteobacteria in the Mackay dataset are surprising outliers, since other studies using the same primer sets (515F-806b for the Heron study and 63F-533R for the Mackay study) did retrieve large numbers of cyanobacterial reads from marine samples collected in the Red Sea[18] and GBR[19], respectively. Still, primer bias or biases in DNA extraction are potential explanations for such differences in community composition at the phyla level, and this again highlights the need for caution when making comparisons across datasets.

There are also obvious limitations in the extrapolation exercise we present to predict microbial communities for the wide-GBR. Firstly, the spatial resolution of the eReefs data obtained, for example, is still coarse (1x1 Km) if compared to the spatial scales at which microbial data was acquired. This means we are necessarily dealing with environmental conditions averaged over large areas, and therefore excluding any heterogeneity that could in fact better relate to the environmental conditions verified at the exact location where the microbial sampling was performed. However, the known congruence in microbial samples retrieved from seawater across sampling locations[16] counterbalances this limitation. Secondly, the range of environmental conditions covered by the LTMP dataset is broader than that covered in the microbial dataset. Even though these ranges overlap (see Supplementary Fig. 1), the microbial sites only cover a minor part of the environmental variation in the GBR benthic categories, which means that the estimated microbial community is only to be found potentially within a shorter range of the full spectrum of environmental variation. This means our estimations are necessarily too constrained and likely there is room to accommodate a broader microbial community.

With regards to the relative importance of the different reef categories across the GBR area mapped (in Fig. 6a), the undisclosed category Out-Tab only represents 1.4% of all mapped GBR reefs. Other outershelf reefs cover about 30% of the GBR (Out-Digit and Out-Soft

accounting for 6.5% and 22.7%, respectively of all mapped reefs). Out-Soft is actually the second largest category, losing only to Mid-Mixed, or midshelf reefs, with 57.5%. Inshore reefs occupy just over 10% of the GBR (In-MA and In-Porites representing 2.3% and 9.7%, respectively, of all mapped GBR reefs). Our microbial predictions thus potentially apply to about 90% of the GBR, but the reality is that much more baseline research is needed until one can have a trustworthy representation of pelagic microbial variation across the wide GBR.

In addition to the longitudinal gradient of environmental and microbial variation, a large latitudinal temperature gradient occurs along the expanse of the GBR. Unfortunately, microbial datasets currently available did not allow robust comparisons to be made across latitudinal scales. The increasing occurrence of the In-Porites category towards the northern sector of the GBR (depicted in Fig. 6a) indicates that a similar bacterial community may be identified at these sites. The positive correlation to temperature also suggests that a slight increase in Synechococcaceae and Rhodobacteraceae may occur. However, there is considerably lower terrestrial run-off in the northern regions of the GBR[20] and so the nutrient levels that drive these changes may not be reached. Brodie et al.[20] reported the absence of a cross-shelf gradient in water quality for the far northern GBR, where mean chlorophyll levels were less than half of those measured for the south and central GBR. This suggests that inshore reef microbial communities could be spatially stable at the northern reef sites and perhaps similar to those found during the wet season in the central GBR (i.e., low chlorophyll levels and high temperature). Establishment of fixed monitoring sites dedicated to sampling and characterizing microbial communities at these spatial and temporal scales are required to test these hypotheses.

Final considerations

The indicator taxa proposed probably consist of specialised lineages that have diversified to occupy a particular niche[21], allowing prediction of the surrounding environment with fairly high confidence. Theoretically, this same indicator approach can be extended to microbial functions, measured either as abundances (or ratios) of particular genes, gene transcripts or even of the proteins they code for[22]. Potentially, many of these indicators will be constituted as indexes or ratios that are able to integrate multiple microbial responses into a unique detection mechanism. It is even suggested that the resolution at which microbiome composition differs among samples may be informative of how phylogenetically conserved the traits under selection are[23]. This means that in the future one may be able to identify microbial indicators not just for prevailing environmental conditions and short-term fluctuations, but also for selective pressures on the reef and shifts therein that may result from loss of ecosystem resilience, for instance. This could facilitate, for instance, the early determination of inshore reefs that are at the turning point between being classified as communities dominated by hard coral communities (In-Porites) and macroalgae communities

(In-MA). While other reef metrics provide this information, there is a recognized inability to accurately measure or predict the role of cumulative impacts across different locations spanning communities with varied susceptibility to runoff-related water quality pressures. Biological indicators have in the past been incorporated into monitoring programs for the GBR as accepted tools to identify the occurrence of environmental stress over space and time. The application of microbial based monitoring and diagnostics is in its infancy, however once established, these approaches will greatly increase our understanding of the biological response of all trophic levels to impacts affecting coral reefs worldwide.

Supplementary References

1 Angly, F. E. *et al.* Marine microbial communities of the Great Barrier Reef lagoon are influenced by riverine floodwaters and seasonal weather events. *PeerJ* **4**, e1511, doi:10.7717/peerj.1511 (2016).

2 Zaneveld, J. R. *et al.* Overfishing and nutrient pollution interact with temperature to disrupt coral reefs down to microbial scales. *Nature Communications* **7**, doi:ARTN 11833
10.1038/ncomms11833 (2016).

3 Zhang, C. L. L., Xie, W., Martin-Cuadrado, A. B. & Rodriguez-Valera, F. Marine Group II Archaea, potentially important players in the global ocean carbon cycle. *Frontiers in Microbiology* **6**, doi:ARTN 1108
10.3389/fmicb.2015.01108 (2015).

4 Iverson, V. *et al.* Untangling Genomes from Metagenomes: Revealing an Uncultured Class of Marine Euryarchaeota. *Science* **335**, 587-590, doi:10.1126/science.1212665 (2012).

5 Frade, P. R., Roll, K., Bergauer, K. & Herndl, G. J. Archaeal and Bacterial Communities Associated with the Surface Mucus of Caribbean Corals Differ in Their Degree of Host Specificity and Community Turnover Over Reefs. *PLoS ONE* **11**, doi:10.1371/journal.pone.0144702 (2016).

6 Thompson, A. *et al.* Marine Monitoring Program. Annual Report for inshore coral reef monitoring: 2016 – 2017. 148 (Townsville, 2018).

7 Alongi, D. M. *et al.* Phytoplankton, bacterioplankton and virioplankton structure and function across the southern Great Barrier Reef shelf. *J. Mar. Syst.* **142**, 25-39 (2015).

8 Berkelmans, R., Weeks, S. J. & Steinberg, C. R. Upwelling linked to warm summers and bleaching on the Great Barrier Reef. *Limnol. Oceanogr.* **55**, 2634-2644, doi:10.4319/lo.2010.55.6.2634 (2010).

9 McCarren, J. *et al.* Microbial community transcriptomes reveal microbes and metabolic pathways associated with dissolved organic matter turnover in the sea. *Proc Natl Acad Sci U S A* **107**, 16420-16427, doi:10.1073/pnas.1010732107 (2010).

10 Bruce, T. *et al.* Abrolhos Bank Reef Health Evaluated by Means of Water Quality, Microbial Diversity, Benthic Cover, and Fish Biomass Data. *PLoS ONE* **7**, doi:ARTN e36687, DOI 10.1371/journal.pone.0036687 (2012).

11 Mizuno, C. M., Rodriguez-Valera, F. & Ghai, R. Genomes of Planktonic Acidimicrobiales: Widening Horizons for Marine Actinobacteria by Metagenomics. *Mbio* **6**, doi:ARTN e02083-14
10.1128/mBio.02083-14 (2015).

12 Haas, A. F. *et al.* Global microbialization of coral reefs. *Nature Microbiology*, 16042, doi:10.1038/NMICROBIOL.2016.42 (2016).

13 Mellin, C. *et al.* Spatial resilience of the Great Barrier Reef under cumulative disturbance impacts. *Global Change Biol.* **25**, 2431-2445, doi:10.1111/gcb.14625 (2019).

14 Brodie, J. E. *et al.* Terrestrial pollutant runoff to the Great Barrier Reef: an update of issues, priorities and management responses. *Mar. Pollut. Bull.* **65**, 81-100 (2012).
15 Wolanski, E., Andutta, F., Deleersnijder, E., Li, Y. & Thomas, C. J. The Gulf of Carpentaria heated Torres Strait and the Northern Great Barrier Reef during the 2016 mass coral bleaching event. *Estuarine Coastal Shelf Sci* **194**, 172-181, doi:10.1016/j.ecss.2017.06.018 (2017).
16 Glasl, B. *et al.* Microbial indicators of environmental perturbations in coral reef ecosystems. *Microbiome* **7**, 94, doi:https://doi.org/10.1186/s40168-019-0705-7 (2019).
17 Brown, M. V. *et al.* Systematic, continental scale temporal monitoring of marine pelagic microbiota by the Australian Marine Microbial Biodiversity Initiative. *Nature Scientific Data* **5**, doi:DOI: 10.1038/sdata.2018.130 (2018).
18 Apprill, A., McNally, S., Parsons, R. & Weber, L. Minor revision to V4 region SSU rRNA 806R gene primer greatly increases detection of SAR11 bacterioplankton. *Aquat. Microb. Ecol.* **75**, 129-137, doi:10.3354/ame01753 (2015).
19 Bourne, D. G. *et al.* Coral reef invertebrate microbiomes correlate with the presence of photosymbionts. *Isme Journal* **7**, 1452-1458, doi:Doi 10.1038/Ismej.2012.172 (2013).
20 Brodie, J., De'ath, G., Devlin, M., Furnas, M. & Wright, M. Spatial and temporal patterns of near-surface chlorophyll a in the Great Barrier Reef lagoon. *Mar Freshw Res* **58**, 342-353, doi:10.1071/Mf06236 (2007).
21 Ngugi, D. K., Blom, J., Stepanauskas, R. & Stingl, U. Diversification and niche adaptations of Nitrospina-like bacteria in the polyextreme interfaces of Red Sea brines. *The ISME journal* **10**, 1383-1399 (2016).
22 Glasl, B., Webster, N. S. & Bourne, D. G. Microbial indicators as a diagnostic tool for assessing water quality and climate stress in coral reef ecosystems. *Mar. Biol.* **164**, doi:Artn 91
10.1007/S00227-017-3097-X (2017).
23 Chase, A. B. & Martiny, J. B. H. The importance of resolving biogeographic patterns of microbial microdiversity. *Microbiology Australia* **38**, 2015-2205, doi:10.1071/MA18003 (2018).
