# Supplementary material for: Spatial patterns of microbial communities across surface waters of the Great Barrier Reef
Source: Commun Biol. 2020 Aug 14;3:442. doi: 10.1038/s42003-020-01166-y (PMC7428009; doi:10.1038/s42003-020-01166-y)
Supplement: Supplementary file 2 — Reporting Summary [file 42003_2020_1166_MOESM2_ESM.pdf]

nature research

Corresponding author(s): Pedro R. Frade

Last updated by author(s): Jul 1, 2020

Reporting Summary

Nature Research wishes to improve the reproducibility of the work that we publish. This form provides structure for consistency and transparency in reporting. For further information on Nature Research policies, see Authors & Referees and the Editorial Policy Checklist.

Statistics

For all statistical analyses, confirm that the following items are present in the figure legend, table legend, main text, or Methods section.

n/a	Confirmed	
☐	☒	The exact sample size (*n*) for each experimental group/condition, given as a discrete number and unit of measurement
☒	☐	A statement on whether measurements were taken from distinct samples or whether the same sample was measured repeatedly
☐	☒	The statistical test(s) used AND whether they are one- or two-sided<br>*Only common tests should be described solely by name; describe more complex techniques in the Methods section.*
☐	☒	A description of all covariates tested
☐	☒	A description of any assumptions or corrections, such as tests of normality and adjustment for multiple comparisons
☐	☒	A full description of the statistical parameters including central tendency (e.g. means) or other basic estimates (e.g. regression coefficient) AND variation (e.g. standard deviation) or associated estimates of uncertainty (e.g. confidence intervals)
☐	☒	For null hypothesis testing, the test statistic (e.g. F, t, r) with confidence intervals, effect sizes, degrees of freedom and P value noted<br>*Give P values as exact values whenever suitable.*
☒	☐	For Bayesian analysis, information on the choice of priors and Markov chain Monte Carlo settings
☐	☒	For hierarchical and complex designs, identification of the appropriate level for tests and full reporting of outcomes
☐	☒	Estimates of effect sizes (e.g. Cohen's *d*, Pearson's *r*), indicating how they were calculated

Our web collection on statistics for biologists contains articles on many of the points above.

Software and code

Policy information about availability of computer code

Data collection	An adapted R script was developed to extract environmental data spanning the period Jan 2015 - Jan 2018 from each 1x1 Km grid cell matching the n=37 microbial sites and n=109 LTMP reference sites which is available here: https://github.com/sammatthews990/eReefs_Fradeetal2019
Data analysis	All meta-analyses of available microbial community composition and contextual environmental data were performed in R version 3.4.3, and graphical outputs were generated with ggplot2

For manuscripts utilizing custom algorithms or software that are central to the research but not yet described in published literature, software must be made available to editors/reviewers. We strongly encourage code deposition in a community repository (e.g. GitHub). See the Nature Research guidelines for submitting code & software for further information.

Data

Policy information about availability of data

All manuscripts must include a data availability statement. This statement should provide the following information, where applicable:

- Accession codes, unique identifiers, or web links for publicly available datasets
- A list of figures that have associated raw data
- A description of any restrictions on data availability

All sequencing data obtained from BioPlatforms Australia (BPA), covering the Burdekin, Yongala and Coral Sea datasets, is available online: https://data.bioplatforms.com/organization/pages/bpa-marine-microbes/
Availability of all other sequencing data (reported in supplementary materials) is detailed in the respective publication for each of the GBR regions: Tully (Angly et al 2016), Mackay (Alongi et al 2015) and Heron Island (Epstein et al 2019).
All environmental data used is available from the eReefs hydrodynamic and biogeochemical model (GBR1, https://research.csiro.au/ereefs/models/model-outputs/gbr1/)

Field-specific reporting

Please select the one below that is the best fit for your research. If you are not sure, read the appropriate sections before making your selection.

☐ Life sciences ☐ Behavioural & social sciences ☒ Ecological, evolutionary & environmental sciences

For a reference copy of the document with all sections, see nature.com/documents/nr-reporting-summary-flat.pdf

Ecological, evolutionary & environmental sciences study design

All studies must disclose on these points even when the disclosure is negative.

Study description	In our manuscript we used an original meta-analysis combining published microbial data with environmental data extracted from a modelling platform to identify the main environmental predictors of microbial community dynamics across surface waters of the Great Barrier Reef. While we initially extracted datasets covering n=167 microbial samples originated from 25 collection sites, the results reported in the main text refer to n=69 microbial samples originated from 10 collection sites and 2 seasons (see sampling strategy and data exclusions below).
Research sample	All sequencing data (reported in the main text) was obtained from BioPlatforms Australia (BPA), covering the Burdekin, Yongala and Coral Sea datasets, and is available online: https://data.bioplatforms.com/organization/pages/bpa-marine-microbes/<br>Availability of all other sequencing data (reported in supplementary materials) is detailed in the respective publication for each of the GBR regions: Tully (Angly et al 2016), Mackay (Alongi et al 2015) and Heron Island (Epstein et al 2019).<br>All environmental data used is available from the eReefs hydrodynamic and biogeochemical model (GBR1, https://research.csiro.au/ereefs/models/model-outputs/gbr1/)
Sampling strategy	Sample sizes were determined by the availability of existing datasets on microbial communities for pelagic environments across the Great Barrier Reef.
Data collection	Microbial data was extracted by Pedro R. Frade from previously published studies. Environmental data was retrieved from the eReefs platform by Samuel A. Matthews using an adapted R script available here: https://github.com/sammatthews990/eReefs_Fradeetal2019
Timing and spatial scale	Environmental data was extracted from the eReefs hydrodynamic and biogeochemical model with spatial and temporal resolution matching the available microbial datasets (and the place/time reported for their collections; spanning 2011-2017). All surface seawater data was extracted using 1x1 Km resolution except for those cases where only the 4x4 Km model could provide data (1x1 Km model only available from 2015 onwards). In all cases, data were averaged across the 3 days leading up to (and including) the actual sampling dates reported in the case studies.
Data exclusions	Comparability between samples was determined by the sequencing/clustering/filtering methods employed by the original studies and was also a criterium used to select the datasets to use in the final analyses. Datasets not useful for comparisons across the wide GBR were nevertheless analyzed (within individual dataset) using the same methods and this is reported in the supplementary materials.
Reproducibility	No experiments were performed. Statistical reproducibility was ensured by using "leave-one-out" cross validation techniques in the case of predictions derived from statistical models, and a high number of permutations was applied to deal with hypothesis-driven statistical tests.
Randomization	Not relevant to this study as we used all previously available datasets for the Great Barrier Reef, to the best of our knowledge.
Blinding	Not relevant to this study as we used all previously available datasets for the Great Barrier Reef, to the best of our knowledge.

Did the study involve field work? ☐ Yes ☒ No

Reporting for specific materials, systems and methods

We require information from authors about some types of materials, experimental systems and methods used in many studies. Here, indicate whether each material, system or method listed is relevant to your study. If you are not sure if a list item applies to your research, read the appropriate section before selecting a response.

Materials & experimental systems

n/a	Involved in the study
☒	☐ Antibodies
☒	☐ Eukaryotic cell lines
☒	☐ Palaeontology
☒	☐ Animals and other organisms
☒	☐ Human research participants
☒	☐ Clinical data

Methods

n/a	Involved in the study
☒	☐ ChIP-seq
☒	☐ Flow cytometry
☒	☐ MRI-based neuroimaging
