# Supplementary material for: Spatial patterns of microbial communities across surface waters of the Great Barrier Reef
Source: Commun Biol. 2020 Aug 14;3:442. doi: 10.1038/s42003-020-01166-y (PMC7428009; doi:10.1038/s42003-020-01166-y)
Supplement: Supplementary file 7 — Description of Additional Supplementary Files [file 42003_2020_1166_MOESM7_ESM.pdf]

DESCRIPTION OF SUPPLEMENTARY DATA FILES

Name: Supplementary Data 1
Description: Figure 2 source data
data_Fig2_new
Environmental variation across the Great Barrier Reef (GBR) for the peaks of the two seasons for parameters retrieved from the eReefs platform, covering several reef locations (Microbial and LTMP) classified into distinct reef categories (Clust), and their geographical coordinates.

Name: Supplementary Data 2
Description: Figure 3a source data
data_Fig3a_new
Diversity indices for microbial communities sampled for several reefs (Microbial sites) across the Great Barrier Reef (GBR)

Name: Supplementary Data 3
Description: Figure 4b source data
data_Fig4_for_suppl.
Average relative abundance of dominant bacterial families across the Great Barrier Reef (GBR) reef categories and for two seasonal peaks.

Name: Supplementary Data 4
Description: Figure 5 source data
data_Fig5a_new
Spearman correlations between individual environmental parameters and the relative abundance of dominant individual bacterial families for the Great Barrier Reef (GBR) and two seasonal peaks, including associated p-value, adjusted p-value and significance of correlation.
